# Hamiltonian engineering of spin-orbit–coupled fermions in a Wannier-Stark optical lattice clock

**DOI:** 10.1126/sciadv.adc9242

**Published:** 2022-10-12

**Authors:** Alexander Aeppli, Anjun Chu, Tobias Bothwell, Colin J. Kennedy, Dhruv Kedar, Peiru He, Ana Maria Rey, Jun Ye

**Affiliations:** ^1^ JILA, National Institute of Standards and Technology, and Department of Physics, University of Colorado, Boulder, CO 80309, USA.; ^2^ Center for Theory of Quantum Matter, University of Colorado, Boulder, CO 80309, USA,

## Abstract

Engineering a Hamiltonian system with tunable interactions provides opportunities to optimize performance for quantum sensing and explore emerging phenomena of many-body systems. An optical lattice clock based on partially delocalized Wannier-Stark states in a gravity-tilted shallow lattice supports superior quantum coherence and adjustable interactions via spin-orbit coupling, thus presenting a powerful spin model realization. The relative strength of the on-site and off-site interactions can be tuned to achieve a zero density shift at a “magic” lattice depth. This mechanism, together with a large number of atoms, enables the demonstration of the most stable atomic clock while minimizing a key systematic uncertainty related to atomic density. Interactions can also be maximized by driving off-site Wannier-Stark transitions, realizing a ferromagnetic to paramagnetic dynamical phase transition.

## Teaser

Interactions in an atomic clock are engineered to eliminate collisional shifts and explore a dynamical phase transition.

## INTRODUCTION

The joint advance of quantum metrology and quantum simulation provides exciting new opportunities to explore the frontiers of measurement science and the emergence of many-body complexity. An outstanding example has been the development of optical lattice clocks (OLCs) where excellent quantum coherence and exquisite quantum control of many atoms have enabled rapid advances in metrological capabilities (*1*–*7*), culminating in the recent demonstration of clock measurement precision at 7.6 × 10^−21^ and near minute-long atomic coherence (*6*). To achieve this level of performance, we use a shallow, vertically aligned optical lattice. The acceleration due to local gravity lifts the degeneracy of neighboring sites, supporting partially delocalized Wannier-Stark (WS) eigenstates. This trapping scheme, first suggested in 2005 (*8*), allows us to operate the clock at substantially smaller lattice depths, greatly suppressing detrimental motional-, light scattering–, and atomic density–induced decoherence.

The use of tilted optical lattices to manipulate motional degrees of freedom in ultracold gases has been widely reported. They have been used to suppress direct tunneling but not spin transport and realize new types of spin Hamiltonians (*9*–*11*), generate spin-orbit coupling (SOC) via laser-assisted tunneling (*12*–*15*), emulate magnetic models in spinless bosons (*16*, *17*), probe nonergodicity due to kinetic constraints (*18*) and subdiffusive transport (*19*) in Fermi-Hubbard chains and many-body localization in trapped ions (*20*), as well as measure gravity in Raman interferometers (*21*–*23*). In this work, we demonstrate how a tilted optical lattice combined with pristine quantum coherence and exquisite spectral resolution offer new capabilities to engineer, drive, and understand many-body systems.

As we continue to push the OLC to new levels of precision, a key remaining issue for clock accuracy is related to frequency shifts associated with atomic interactions. Quantum statistics dictates that identical fermions experience only odd partial wave interactions that are suppressed at ultralow temperatures (*24*–*29*). Yet, even the weak elastic and inelastic p-wave collisions were found to substantially affect clock operation and limit the number of interrogated atoms at deep lattice depths. As atoms delocalize along neighboring sites in the shallow lattice, p-wave collisions are reduced but s-wave interactions can emerge from the SOC generated by the differential clock laser phase (*30*–*32*). The superior quantum coherence obtained in our gravity-tilted OLC stems from better control over motional and internal degrees of freedom (*6*), allowing the engineering of s- and p-wave interactions in driven spin-orbit–coupled fermionic atoms. By operating at the “magic” lattice depth where s-wave interactions precisely cancel residual p-wave interactions, we reduce atomic-interaction induced shifts in our one-dimensional (1D) lattice clock to a fractional frequency shift of 5.0 (1.7) × 10^−21^ per atom at a single site.

We further explore the tunability of atomic interactions by driving a site-changing WS transition. This leads to an atomic superposition that not only carries a distinct internal label but also features different motional orbitals. As a consequence, s-wave interactions are substantially enhanced. This gives rise to a many-body dynamical phase transition (DPT) between dynamical ferromagnetic and paramagnetic states controlled by the interplay between the clock drive and atomic interactions. Although similar DPTs have been observed in trapped ions (*33*), superconducting qubits (*34*), and atoms in cavities (*35*) and optical traps (*36*), here, we use in situ imaging to locally resolve the emergence of a nonlinear excitation lineshape as a function of atom number.

## RESULTS

### System in consideration

Several hundreds of thousands of nuclear spin-polarized fermionic ^87^Sr atoms are cooled via standard techniques and loaded into a vertical 1D optical lattice that defines the $\hat{Z}$ axis (*6*). We load the lattice at a depth of 300 lattice photon recoil energies (*E*_rec_) with atoms in the lowest motional band along the $\hat{Z}$ axis. Perpendicular to the lattice axis, the atoms are weakly confined and thermally populate the resultant radial modes with a temperature of 800 nK. We then adiabatically reduce the lattice depth to a much lower operational depth with a correspondingly reduced radial temperature measured with Doppler spectroscopy.

**Fig. 1.**
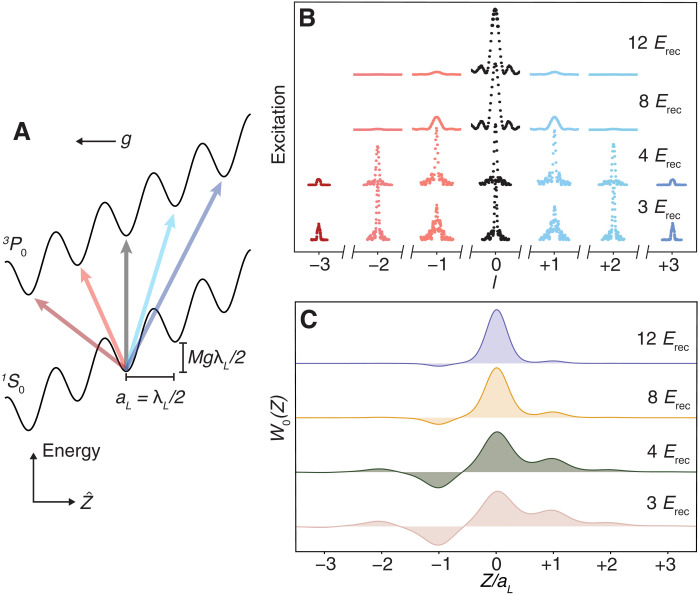
The WS clock. (**A**) We trap ^87^Sr atoms in a 1D optical lattice along the $\hat{Z}$ direction aligned with local gravitational acceleration *g*. This type of external confinement realizes WS states, eigenstates of the joint lattice, and the gravitational potential. The *n*-th WS state *W_n_*(*Z*) is centered at lattice site *n* and has energy *Mga_L_n*, where *M* is the mass of ^87^Sr and *a_L_* = λ*_L_*/2 is the lattice spacing with lattice wavelength λ*_L_*. The WS ladder creates a set of transitions from the ground (∣*g*〉 ≡ ∣^1^*S*_0_, *m_F_* = ± 5/2〉) to clock (∣*e*〉 ≡ ∣^3^*P*_0_, *m_F_* = ± 3/2〉) state at different lattice sites accessible by the differential clock laser phase between them. The black line indicates a carrier ∣*g*; *W_n_*〉 → ∣*e*; *W_n_*〉 transition. At shallow lattice depths, a set of off-site transitions ∣*g*; *W_n_*〉 → ∣*e*; *W*_*n*±*l*_〉 for integer *l* are indicated by blue and red lines. (**B**) At shallow lattice depths, the atomic wave function becomes delocalized, allowing ∣*g*; *W_n_*〉 → ∣*e*; *W*_*n*±*l*_〉 transition for a range of *l* to be addressed. Here, we show Rabi scans of these transitions at four different lattice depths, given in lattice photon recoil energy (*E*_rec_). As the depth decreases, the Rabi frequency on the carrier transition decreases. We correspondingly lengthen the pulse time to maintain a π pulse on the carrier leading to narrower lineshapes at shallow depths. (**C**) The wave function *W*_0_(*Z*) for the four corresponding lattice depths, illustrating the tunable delocalization due to the interplay between lattice and gravitational potential.

The gravitational potential with local acceleration *g* adds a linear energy gradient across the lattice, with the combined single-particle Hamiltonian supporting WS eigenstates. The WS state *W_n_*(*Z*) is centered at lattice site *n* and has eigenenergy *Mga_L_n*, where *M* is the mass of ^87^Sr and *a_L_* = λ*_L_*/2 is the lattice site spacing (Fig. 1A). Here, we use the strontium magic wavelength λ*_L_* = 813 nm, guaranteeing identical confinement for both clock states.

The clock laser λ*_c_* = 698 nm, aligned along the lattice, drives the ultranarrow ∣^1^*S*_0_, *m_F_* = ± 5/2〉→∣^3^*P*_0_, *m_F_* = ± 3/2〉 (∣*g*〉 → ∣*e*〉) clock transition, where *m_F_* is the nuclear Zeeman level. This σ-polarized transition is the least magnetically sensitive clock transition in ^87^Sr. Because the clock laser wavelength differs from the lattice spacing, adjacent lattice sites see a different clock phase φ = πλ*_L_*/λ*_c_* ≈ 7π/6. This phase difference generates SOC when the lattice depth is sufficiently low for atoms to tunnel during the course of the experiment. Thus, when tuned to appropriate frequencies, the clock laser effectively couples WS states between different lattice sites, i.e., ∣*g*; *W_n_*〉 → ∣*e*; *W*_*n*+*l*_〉, for a range of integer *l*. The corresponding Rabi frequency Ω*_l_* set by the wave function overlap is

$$\Omega_{l}\propto \text{exp}\;(-\frac{\lambda_{L}^{2}}{4\lambda_{c}^{2}\sqrt{V_{0}}})J_{l}(\frac{4J_{0}}{\mathrm{Mg}a_{L}}\text{sin}\;(\varphi /2))$$ (1)

Here, 𝒥*_l_* is a Bessel function, *J*_0_ is the nearest-neighbor tunneling energy of the ground band, and *V*_0_ is the lattice depth in *E*_rec_.

We use Rabi spectroscopy in a dilute ensemble to demonstrate the partially delocalized nature of the single-particle wave functions in shallow, tilted lattices of four different values of *V*_0_, shown in Fig. 1B. The corresponding WS wave functions *W*_0_(*Z*) are shown in Fig. 1C. For each *V*_0_, we optimize the transition probability on the carrier transition, ∣*g*; *W_n_*〉 → ∣*e*; *W_n_*〉. For *V*_0_ = 12 *E*_rec_, the atoms are still well localized, and thus, the ∣*g*; *W_n_*〉 → ∣*e*; *W*_*n*±1_〉 transition amplitudes are substantially suppressed in comparison to the carrier. As *V*_0_ is reduced, we resolve a set of Rabi lines spectrally separated by *Mga_L_*/*h* = 867 Hz, where *h* is Planck’s constant. At 4 *E*_rec_, the Rabi frequency for the carrier and ∣*g*; *W_n_*〉 → ∣*e*; *W*_*n*±1_〉 transitions are roughly equivalent. At 3 *E*_rec_, the carrier and ∣*g*; *W_n_*〉 → ∣*e*; *W*_*n*±2_〉 have similar Rabi frequencies, while the ∣*g*; *W_n_*〉 → ∣*e*; *W*_*n*±1_〉 transition has the greatest Rabi frequency and is thus overdriven. At low atomic density, we observe coherence times well past 10 s on ∣*g*; *W_n_*〉 → ∣*e*; *W*_*n*+1_〉 (see the Supplementary Materials).

### Theoretical model

Under our operating conditions, where the collisional rate for motional relaxation is smaller than the internal spin dynamics and trap frequencies, atoms remain effectively frozen in single-particle eigenstates during clock interrogation. Because all atoms are initially prepared in a single internal state, Fermi statistics forbids double occupancy of motional states. Under these conditions, the quantum dynamics can be described with a spin Hamiltonian in energy space spanned by the appropriate single-particle trap eigenmodes (*28*, *29*, *36*–*38*). We identify a two-level system for an atom in mode **n** as∣↑_**n**_〉 ≡ ∣*e*; *n_X_*, *n_Y_*, *W_n_*〉 and ∣↓_**n**_〉 ≡ ∣*g*; *n_X_*, *n_Y_*, *W_n_*〉. Here, *n_X_* and *n_Y_* label the radial harmonic oscillator modes.

Two dominant types of interatomic interactions determine the coupling constants in the spin model: local interactions between atoms within a single lattice site and nearest-neighbor interactions between atoms in adjacent sites. Next to nearest-neighbor interactions are on the order of 10^−2^ of nearest-neighbor interactions or smaller for the operating conditions in our system and are neglected. The couplings between radial harmonic oscillator modes are highly collective as shown in previous experiments (*28*, *29*, *37*). Therefore, to an excellent approximation, we define collective spin operators at each lattice site after summing over occupied harmonic oscillator modes, ${\hat{S}}_{n}^{x,y,z}=\sum_{n_{X},n_{Y}}{\hat{S}}_{n}^{x,y,z}$. The dynamics of the collective spin vector $\langle {\hat{S}}_{n}\rangle =\{\langle {\hat{S}}_{n}^{x}\rangle ,\langle {\hat{S}}_{n}^{y}\rangle ,\langle {\hat{S}}_{n}^{z}\rangle \}$ are described by the following mean-field equation of motion written in a gauge frame where the laser drive is homogeneous (see the Supplementary Materials)

$$\frac{d}{\mathrm{dt}}\langle {\hat{S}}_{n}\rangle =B^{⊥}\times \langle {\hat{S}}_{n}\rangle$$ (2)

The synthetic magnetic field **B**^⊥^ contains contributions of the laser drive with detuning δ from the bare transition and the self-generated interactions terms

$$B^{⊥}=\{\Omega_{0},0,-\delta +2(\chi_{0}+\chi_{1})\langle {\hat{S}}^{z}\rangle +C_{0}N_{\text{loc}}\}$$ (3)

Here, $\langle {\hat{S}}^{z}\rangle =\frac{1}{2L+1}\sum_{m=-L}^{L}\langle {\hat{S}}_{n+m}^{z}\rangle$ is the average magnetization over a region of 2*L* + 1 ∼ 15 lattice sites (corresponding to 1 camera pixel or 6 μm in our imaging spectroscopy) centered around *n*. *N*_loc_ is the number of atoms per lattice site averaged over the same region. The couplings, χ_0_ = η_0_(*V_ee_* + *V_gg_* − 2*V_eg_*)/2, *C*_0_ = η_0_(*V_ee_* − *V_gg_*)/2 and χ_1_ = − η_1_*U_eg_*(1 − cos φ), respectively, describe thermally averaged p-wave and s-wave interaction parameters between internal clock states as well as on the on-site (η_0_) and nearest-neighbor (η_1_) overlap matrix elements along the lattice. In the absence of SOC, φ = 0, the s-wave interactions vanish.

Without interactions, the collective spin features a characteristic Rabi lineshape profile when driven during a pulse area Ω_0_*T* = π with an excitation fraction $n_{↑}(t)=\langle {\hat{S}}^{z}(t)\rangle /N_{\text{loc}}+1/2$, symmetric and centered around δ = 0. With interactions, the time evolution takes place in the presence of an additional self-generated axial magnetic field–like term that induces a nonlinear response, resulting in an asymmetric lineshape. A simple estimation of the density shift can be obtained by setting it to be the value of δ at which $B_{z}^{⊥}=0$

$$\Delta \nu_{\alpha \to \beta }=\Delta \nu_{\alpha \to \beta }^{s}+\Delta \nu_{\alpha \to \beta }^{p}$$ (4)

$$2\pi \Delta \nu_{\alpha \to \beta }^{p}\approx 2\chi_{0}\varsigma_{\alpha \to \beta }^{z}+C_{0},\;\;2\pi \Delta \nu_{\alpha \to \beta }^{s}\approx 2\chi_{1}\varsigma_{\alpha \to \beta }^{z}$$ (5)

Here, $\Delta \nu_{\alpha \to \beta }^{s,p}$ are the s-wave and p-wave contributions to the density shift, and α and β indicate initial and final states, ∣*g*〉 or ∣*e*〉. $\varsigma_{\alpha \to \beta }^{z}$ is a fitting parameter that accounts for the time evolution of $\langle {\hat{S}}^{z}\rangle /N_{\text{loc}}$ during the Rabi dynamics, which depends on the details of the Rabi drive such as the pulse area, excitation fraction, and initial conditions used in the experiment (see the Supplementary Materials).

### Density shifts in the carrier transition

To measure the effect of collisional shifts on the clock transition, we perform extended measurements using a “clock lock” to track the drift of the laser. Each clock lock consists of a set of four lock points, a standard interleaved sequence probing opposite sign *m_F_* states to reject first-order Zeeman shifts. As reported in (*6*), we use in situ imaging to construct a microscopic frequency map throughout the extended sample, fitting a linear slope to the relationship between frequency and number of atoms per site at each lock point. We define a linear density shift coefficient Δν_α→β_/ν such that the total fractional frequency shift is the product of this coefficient and *N*_loc_, calibrated using quantum projection noise techniques. The reported values of Δν_α→β_/ν are the weighted mean of Δν_α→β_/ν at every lock point during an extended clock lock measurement campaign. The statistical uncertainty is given by the Allan deviation fit at one-sixth total measuring time.

**Fig. 2.**
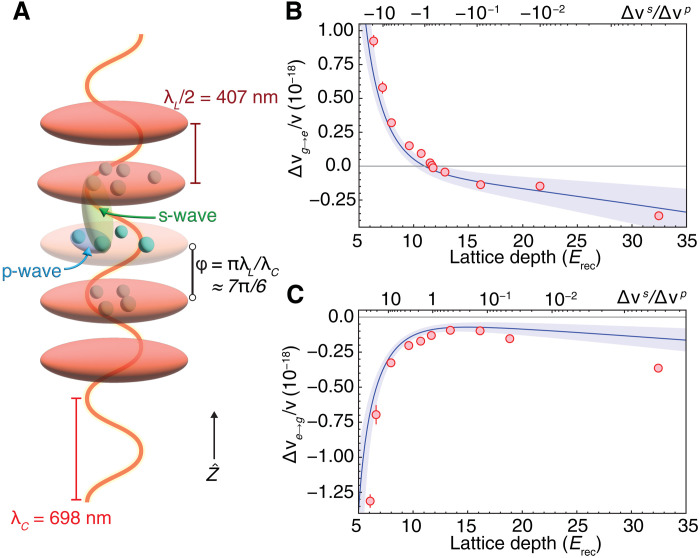
Engineering interactions. (**A**) By varying the lattice depth during clock spectroscopy, we modify the ratio of off-site s-wave to on-site p-wave collisional shifts, $\Delta \nu_{\alpha \to \beta }^{s}/\Delta \nu_{\alpha \to \beta }^{p}$, where α and β indicate clock states. Atoms are trapped in an optical lattice with wavelength λ*_L_* and probed by clock light with wavelength λ*_C_*. Each antinode of the lattice light traps a number of atoms that interact via p-wave collisions. The 698-nm clock wavelength is incommensurate with the lattice spacing, so atoms in neighboring lattice sites see different clock phases, φ = πλ*_L_*/λ*_C_* ≈ 7π/6, allowing s-wave interactions at low lattice depths. (**B**) The fractional frequency density shift Δν_*g*→*e*_/ν over a range of lattice depths. Red points and error bars indicate experimental data and corresponding uncertainty in density shift and lattice depth. The theoretical density shift is shown as a solid blue line with the shaded blue region accounting for uncertainties in the s-wave scattering length and p-wave scattering volumes (*37*, *41*), as well as 10 nK temperature uncertainty. (**C**) The density shift Δν_*e*→*g*_/ν over a range of lattice depths.

In Fig. 2, we plot the measured coefficients over a range of *V*_0_ for both the ∣*g*〉 → ∣*e*〉 and ∣*e*〉 → ∣*g*〉 transition. We typically use a 3.2 s π pulse duration. To account for increased delocalization and reduced Rabi frequencies at the shallowest depths, we lengthen the pulse time. The effect of s-wave collisions at low lattice depths is readily apparent, with a marked increase in density shift between 12 and 5 *E*_rec_, consistent with the growth of the off-site matrix element η_1_ as *V*_0_ is reduced. For the ∣*g*〉 → ∣*e*〉 transition presented in Fig. 2B, the s-wave frequency shift has an opposite sign compared to that of the p-wave. At the magic lattice depth, the s-wave and p-wave shifts have the same magnitude, resulting in a nearly perfect cancellation for a vanishingly small collisional frequency shift. In the ∣*e*〉 → ∣*g*〉 case presented in Fig. 2C, the s-wave frequency shift has the same sign as that of the p-wave, and thus, the density shift remains negative over all lattice depths. This behavior is well described by the mean-field solution from Eq. 2, represented by the solid blue lines in Fig. 2B and Fig. 2C. The disagreement at a large *V*_0_ of 32 *E*_rec_, as shown in Fig. 2C, likely arises from lattice photon–assisted excited state decay to other spin states, leading to background s-wave interactions not included in our theoretical model.

**Fig. 3.**
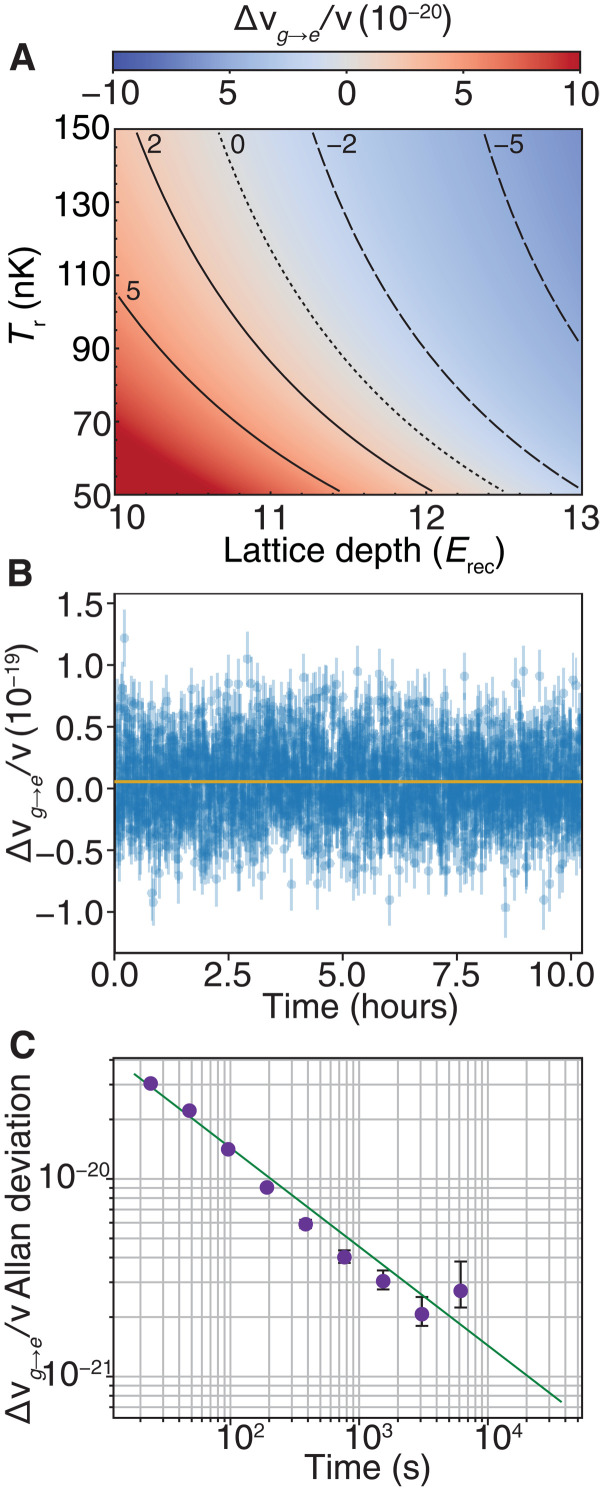
Density shift cancelation. (**A**) At 11.7(0.1) *E*_rec_, the contributions from p-wave and s-wave interactions balance, allowing clock operation with a density shift many orders lower than previous regimes. The heatmap and contours show the calculated fractional frequency shift coefficient Δν_*g* → *e*_/ν for a range of radial temperatures *T*_r_ and lattice depths in our system. (**B**) Over a 10-hour measurement, we report a mean coefficient $\Delta \nu_{g\to e}/\nu =5.0(1.7)\times 10^{-21}$. For each set of four lock points, we extract a density shift coefficient, shown with corresponding uncertainty in blue. The weighted mean over the duration of the run is in gold. (**C**) Allan deviation of the density shift coefficient (purple dots) with corresponding uncertainty reported in error bars. The green line is an instability fit with slope $1.3\times 10^{-19}/\sqrt{\tau }$ for averaging time τ.

In Fig. 3A, we model the fractional frequency shift over a range of experimentally relevant lattice depths and radial temperatures near this magic point. The density shift is sensitive to ensemble temperature, lattice depth, and excitation fraction. Experimentally, the lattice depth is maintained through a precise and large bandwidth lattice intensity servo, and our clock lock tracks the laser drift to ensure a similar excitation fraction throughout the measurement duration. The atomic temperature is less precisely controlled, with small drifts in the cooling laser frequency and stray magnetic fields contributing to reduced cooling reproducibility and observed 10 nK variation. To evaluate the robustness of operating at the magic lattice depth, we demonstrate a 10-hour clock lock using a 3.2 s Rabi probe near the magic depth and report a 5.0 (1.7) × 10^−21^ fractional frequency shift per atom, as shown in Fig. 3B. There is no apparent long-term trend in the density shift, and the coefficient seems to reach a flicker beyond ∼1000 s, as shown by the Allan deviation in Fig. 3C.

The data presented in Fig. 3 was collected with an average of 51 atoms per site in the studied region. For comparison, the synchronous measurement presented in (*6*) with single-clock instability of 3.1 × 10^−18^ at 1 s used 0.5 mm length samples with an average of 38 atoms per site. Operating in the density shift regime near the magic lattice depth presented here, the average density shift magnitude would be approximately 1.9 (0.6) × 10^−19^.

### Dynamical phase transition

**Fig. 4.**
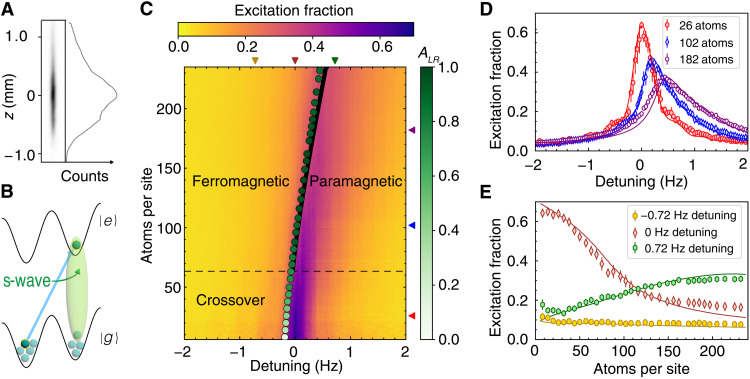
Dynamical phase transition. (**A**) An image of lattice trapped atoms, indicating a spatial extent over a millimeter in length. Within a single image, we can study lattice site density regimes ranging over two orders of magnitude, shown here as camera counts. (**B**) Addressing the ∣*g*; *W_n_*〉 → ∣*e*; *W*_*n*+1_〉 transition, s-wave interactions effectively become on-site, leading to a strong collisional shift. (**C**) Excitation fraction as a function of detuning and atom number on the ∣*g*; *W_n_*〉 → ∣*e*; *W*_*n*+1_〉 transition at 22 *E*_rec_. Above ~63 atoms per site, denoted by the dashed black line, the system features a DPT between ferromagnetic and paramagnetic phases when varying the laser detuning and atomic density. The phase boundary is denoted by a solid black line from theoretical calculations and green points from the experimental data. The normalized asymmetry of the lineshape *A_LR_* is indicated by the shade of these points. Arrows on the right and top axis indicate data plotted in (D) and (E) at constant atom number and detuning. (**D**) Excitation fraction as a function of detuning at different atom numbers demonstrates the notable distortion and asymmetry that arises in the strongly interacting regime. (**E**) Excitation fraction as a function of atom number in the ferromagnetic phase (−0.72 Hz), across the phase transition (0 Hz), and in the paramagnetic phase (0.72 Hz). Solid lines in (D) and (E) indicate theoretical calculations.

By addressing a transition to a different WS state, we further modify the atomic interactions. We can still define an interaction spin model by identifying the states ∣↑_**n**_〉 ≡ ∣*e*; *n_X_*, *n_Y_*, *W*_*n*+*l*_〉 and ∣↓_**n**_〉 ≡ ∣*g*; *n_X_*, *n_Y_*, *W_n_*〉 as the spin-1/2 internal levels. In particular, we interrogate the *l* = 1 transition. The many-body dynamics are then described by the same mean field equation of motion, Eq. 2, but with a different effective magnetic field (see the Supplementary Materials)

$$B_{l=1}^{⊥}\approx \{\Omega_{1},0,-\delta_{1}+2\chi_{1}^{l=1}\langle {\hat{S}}^{z}\rangle \}$$ (6)

where $\chi_{1}^{l=1}=-\eta_{0}U_{\mathrm{eg}}/2$ and δ_1_ is the detuning of the laser to the *l* = 1 transition. Note that because the wave function of the excited state is displaced by one lattice site (see Fig. 4B), the overlap matrix element that characterizes the s-wave interactions is proportional to η_0_. Therefore, atomic interactions are substantially enhanced in this case and increase with higher trap depth. Although the SOC phase does not enter directly in $\chi_{1}^{l=1}$, SOC still plays a key role by allowing the transition to be driven (see Eq. 1). The stronger interactions modify the spin dynamics more markedly and give rise to a DPT between dynamical ferromagnetic and paramagnetic phases (see the Supplementary Materials). The DPT appears as a sharp change in behavior of the long-time average excitation fraction for an initial state prepared with all atoms in ∣*g*〉, $\bar{n_{↑}}=\underset{T\to \infty }{\text{lim}}\frac{1}{T}\int_{0}^{T}n_{↑}(t)dt$. In the dynamical ferromagnetic phase, interactions dominate and the system features small oscillations near a single pole of the Bloch sphere, with $\bar{n_{↑}}\approx 0$. In the dynamical paramagnetic phase, the system exhibits large excursions around the Bloch sphere, and $\bar{n_{↑}}$ dynamically adjusts itself as δ_1_ is varied. In the interaction dominant regime, the DPT generates a second order critical line that distinguishes the two dynamical phases. The transition evolves into a smooth crossover region in the weakly interacting regime, where the dynamics are dominated by single-particle Rabi flopping.

Similar to other DPT experiments, instead of direct measurements of $\bar{n_{↑}}$, the order parameter is estimated by measuring the excitation fraction at a fixed probe time. We use a 2.3 s Rabi π pulse with lattice depth *V*_0_ = 22 *E*_rec_ and radial temperature *T*_r_ = 190 nK. Within a single image, we observe a density range spanning over two orders of magnitude (Fig. 4A). We spatially resolve the excitation fraction within the sample and construct the dynamical phase diagram shown in Fig. 4C.

For a given *N*_loc_, we extract the lineshape asymmetry *A_LR_* defined as (*n_R_* − *n_L_*)/(*n_R_* + *n_L_*) from experimental data and normalize by the maximum value of *A_LR_*. Here, $n_{R}=\int_{\delta_{\text{max}}}^{\delta_{\text{max}}+f}n_{↑}(\delta )d\delta$, $n_{L}=\int_{\delta_{\text{max}}-f}^{\delta_{\text{max}}}n_{↑}(\delta )d\delta$, where δ_max_ is the detuning for the peak value of the Rabi lineshape, and *f*/2π = 1 Hz covers almost the entire frequency range of the Rabi lineshape. The lineshape asymmetry allows us to characterize the dynamical phases. For *N*_loc_ < 63, below the dashed black line in Fig. 4C, the system is in a crossover regime featuring a linear density shift and asymmetry *A_LR_* that becomes more pronounced as the atom number increases. More than 63 atoms per site, the lineshape is near maximally asymmetric, and distinct ferromagnetic and paramagnetic dynamical phases are identified. The phase boundary is experimentally determined by finding the maximum derivative of the lineshape as a function of detuning, plotted as green points in Fig. 4C, with *A_LR_* indicated by the shade. The points lie very close to the theoretically calculated phase boundary shown as a solid black line.

The asymmetry in the lineshape becomes apparent by viewing the excitation at a constant atom number, as in Fig. 4D. At densities well below the crossover boundary, the lineshape is only slightly distorted from that of an ideal Rabi response. Above the crossover density, the excitation displays very different behaviors for the two opposite signs of detuning, and the excitation becomes highly insensitive to changes of detuning deep in the ferromagnetic phase. The constant detuning profiles presented in Fig. 4E further illustrate this DPT. At δ_1_/2π = 0 Hz, the laser drive is on resonance with the noninteracting transition. Above the crossover regime, the ensemble features both dynamical phases, evolving from a dynamical paramagnet to a dynamical ferromagnet for *N*_loc_ > 82. At δ_1_/2π = − 0.72 Hz, the system is in the dynamical ferromagnetic phase above the crossover region. However, with δ_1_/2π = 0.72 Hz detuning, the excitation fraction initially rises with atom number when the system is in the paramagnetic phase and saturates close to the phase boundary. In Fig. 4 (D and E), the solid lines are theoretical predictions from the mean-field spin model with an additional dephasing term accounting for mode-changing collisions (see the Supplementary Materials).

## DISCUSSION

Operating in the WS regime has realized a new and optimized platform for OLCs, with record coherence time and clock precision (*6*). The work here highlights the use of Hamiltonian engineering and control of atomic interactions to remove the compromise between increased precision and reduced systematic uncertainties. Operating with hundreds of thousands of atoms, we still limit the density-related frequency shift well below the current state of the art, and further reduction in density shift is readily attainable. This work uses precise tuning of interactions to explore rich many-body behavior. With selective WS interrogation and in situ imaging, we efficiently map out a DPT over a range of density of more than two orders of magnitude.

So far, we operate in a regime where a mean-field model is sufficient to describe the many-body dynamics. Driving the system with more sophisticated pulse sequences will allow us to further explore quantum correlation and beyond mean-field effects. This will open a path for the generation of spin-squeezed states with a net quantum metrological advantage for state-of-the-art quantum sensors.

## MATERIALS AND METHODS

The experimental apparatus and many of the sample preparation techniques were previously described in (*6*). We prepare a nuclear spin-polarized, cold sample of ^87^Sr at a high lattice depth of 300 *E*_rec_ and then adiabatically reduce the lattice depth. To ensure the atoms are in the lowest motional band along the lattice (axial) direction, we sideband cool on the ^1^*S*_0_ → ^3^P_1_ transition and probe the mode filling using sideband spectroscopy on the clock transition. With sideband cooling, the red sideband is entirely eliminated, indicating sample preparation in the lowest axial motional state. Characteristic sideband spectra are shown in the Supplementary Materials. The absence of motional excitation is confirmed after lattice ramping to the operational depth.

As in (*39*), we fit the sideband spectra to extract a lattice depth. At very low lattice intensities, this fitting technique is no longer a reliable method to determine the lattice depth. Instead, we use the transmitted lattice power and depth fits at higher intensities to calculate the lattice depths at our operational points.

In addition to axial sideband cooling, we use low-field Doppler cooling to reduce the radial temperature. We measure the radial temperature *T*_r_ of the ensemble before each density shift measurement by driving the narrow clock transition with a beam oriented perpendicular to the lattice direction and extracting a Doppler absorption profile. Our camera-based imaging spectroscopy technique provides a spatial map of the temperature throughout the millimeter-length cloud. We observe temperature variations of up to 10 nK over the entire sample. The temperature over the range of operational lattice depths *V*_0_ is well described by

$$T_{r}(\text{nK})=\{\begin{array}{c} -45.2+14.1V_{0}/E_{\text{rec}}\;(V_{0}<15E_{\text{rec}})\\ 42\sqrt{V_{0}/E_{\text{rec}}}\;(V_{0}>15E_{\text{rec}}) \end{array}$$ (7)

For *V*_0_ > 15 *E*_rec_, the trend of *T_r_* matches that expected from an adiabatic lowering of the trap depth. At sufficiently low lattice depths, the anharmonic radial trap behavior leads to a deviation from adiabatic temperatures. For the lowest values of *T*_r_ approaching 20 nK, our Doppler spectroscopy technique also reaches its limit of reliability.

We use extended clock locks to measure the spatially dependent average density shift. For each experimental cycle consisting of four Rabi probes, we construct a frequency map throughout the sample using in situ imaging. The total number of counts at each pixel is proportional to the number of atoms, calibrated using the standard quantum projection noise techniques (*40*). The four laser-frequency lock points probe opposite sign *m_F_* transitions. From the mean frequency of these two transitions, we find the transition frequency at each pixel. We fit this frequency as a function of atom number with a linear model, weighting by the atom number to account for quantum projection noise. The slope of this fit is the density shift coefficient for one experimental cycle. For each lattice depth, the density shift coefficient reported in the main text is the mean of all the coefficients measured over an extended clock lock sequence, with the uncertainty arising from an Allan deviation of these coefficients. We noticed coefficient flicker at long measurement times for the stronger density shift regimes, and thus we quote the uncertainty at one-sixth the measurement time for all configurations.

## Supplementary Materials

### This PDF file includes:

Sections S1 to S4 Figs. S1 to S7 Table S1 References

## Notes

View/request a protocol for this paper from *Bio-protocol*.
