# Supplementary material for: Hamiltonian engineering of spin-orbit–coupled fermions in a Wannier-Stark optical lattice clock
Source: Sci Adv. 2022 Oct 12;8(41):eadc9242. doi: 10.1126/sciadv.adc9242 (PMC9555777; doi:10.1126/sciadv.adc9242)
Supplement: Supplementary file 1 — Sections S1 to S4 Figs. S1 to S7 Table S1 References [file sciadv.adc9242_sm.pdf]

Science Advances
AAAS

Supplementary Materials for

Hamiltonian engineering of spin-orbit–coupled fermions in a Wannier-Stark optical lattice clock

Alexander Aeppli *et al.*

Corresponding author: Jun Ye, ye@jila.colorado.edu; Ana Maria Rey, arey@jilau1.colorado.edu

Sci. Adv. **8**, eadc9242 (2022)
DOI: 10.1126/sciadv.adc9242

The PDF file includes:

Sections S1 to S4
Figs. S1 to S7
Table S1
Legend for data file S1
References

Other Supplementary Material for this manuscript includes the following:

Data file S1

S1 Theoretical Model

S1.1 SU(N) interactions in the Sr optical lattice clock

Fermionic ^{87}Sr atoms have two long-lived electronic orbitals, the $^1S_0$ and $^3P_0$ clock states, as well as a nuclear spin degree of freedom with $I = 9/2$. We denote the electronic states as $|g\rangle$ and $|e\rangle$ respectively and the $N = 2I + 1$ nuclear spin levels as $m = -I, -I + 1, \cdots, I$. Due to the lack of hyperfine coupling between the nuclear and electronic degrees of freedom, the scattering parameters that describe two-body interactions are independent of the nuclear spin states. This property gives rise to an interaction Hamiltonian invariant under SU(N) rotations (*37, 42*).

S-wave interactions occur under spatially symmetric collisions. Due to the requirement for fermionic atoms to feature a fully antisymmetric total wave function, to collide under the s-wave channel symmetric nuclear spin states require their electronic orbitals to be antisymmetric. Therefore, the state $(|ge\rangle - |eg\rangle)/\sqrt{2}$ is the only one that can feature s-wave interactions, characterized by the s-wave scattering length a_{eg}^-. Similarly, to collide via s-wave interactions, antisymmetric nuclear spin states require their electronic orbitals to be symmetric. So there are three possible combination of electronic states $|gg\rangle$, $|ee\rangle$, $(|ge\rangle + |eg\rangle)/\sqrt{2}$ that can collide via s-wave. Their interactions are characterized by the s-wave scattering lengths a_{gg}, a_{ee} and a_{eg}^+ respectively.

Defining the permutationally symmetric ($\mathcal{P}_+$) and permutationally antisymmetric ($\mathcal{P}_-$) projector operators, $\mathcal{P}_\pm = (\mathcal{I} \pm \mathcal{P}_{12})/2$ with $\mathcal{P}_{12} = \sum_{mm'} |m\rangle_1\langle m'| \otimes |m'\rangle_2\langle m|$, where m, m' label nuclear spin levels, the resulting s-wave pseudopotential for SU(N) interaction takes the following form: $V_s^{gg}(\mathbf{R}_{12}) \propto a_{gg}\mathcal{P}_-$, $V_s^{ee}(\mathbf{R}_{12}) \propto a_{ee}\mathcal{P}_-$, $V_s^{eg}(\mathbf{R}_{12}) \propto (a_{eg}^- + a_{eg}^+)\mathcal{I}/2 + (a_{eg}^- - a_{eg}^+)\mathcal{P}_{12}/2$. Thus the s-wave interaction Hamiltonian in the second

quantized form,

$$
\begin{aligned}
H_s = &\frac{2\pi\hbar^2 a_{gg}}{M} \sum_{\substack{mm' \\ (m\neq m')}} \int \mathrm{d}^3\mathbf{R}\, \psi^\dagger_{gm}(\mathbf{R})\psi^\dagger_{gm'}(\mathbf{R})\psi_{gm'}(\mathbf{R})\psi_{gm}(\mathbf{R}) \\
&+ \frac{2\pi\hbar^2 a_{ee}}{M} \sum_{\substack{mm' \\ (m\neq m')}} \int \mathrm{d}^3\mathbf{R}\, \psi^\dagger_{em}(\mathbf{R})\psi^\dagger_{em'}(\mathbf{R})\psi_{em'}(\mathbf{R})\psi_{em}(\mathbf{R}) \\
&+ \frac{2\pi\hbar^2 (a^-_{eg}+a^+_{eg})}{M} \sum_{mm'} \int \mathrm{d}^3\mathbf{R}\, \psi^\dagger_{gm}(\mathbf{R})\psi^\dagger_{em'}(\mathbf{R})\psi_{em'}(\mathbf{R})\psi_{gm}(\mathbf{R}) \\
&+ \frac{2\pi\hbar^2 (a^-_{eg}-a^+_{eg})}{M} \sum_{mm'} \int \mathrm{d}^3\mathbf{R}\, \psi^\dagger_{gm}(\mathbf{R})\psi^\dagger_{em'}(\mathbf{R})\psi_{em}(\mathbf{R})\psi_{gm'}(\mathbf{R}),
\end{aligned}
\tag{S1}
$$

where M is the mass of a Sr atom, $\psi_{gm}(\mathbf{R})$ and $\psi_{em'}(\mathbf{R})$ are fermionic annihilation field operators of nuclear spin m in ground manifold and nuclear spin m' in excited manifold respectively.

For spatially antisymmetric p-wave interactions, antisymmetric nuclear spin states require their electronic orbitals to be antisymmetric for an antisymmetric total wavefunction, so the only possible electronic state is $(|ge\rangle - |eg\rangle)/\sqrt{2}$, which interacts via the p-wave scattering volume $(b^-_{eg})^3$. Symmetric nuclear spin states require their electronic orbitals to be symmetric, so the three possible electronic states are $|gg\rangle$, $|ee\rangle$, $(|ge\rangle + |eg\rangle)/\sqrt{2}$, which interact via the p-wave scattering volumes b^3_{gg}, b^3_{ee} and $(b^+_{eg})^3$ respectively. The p-wave pseudopotential for SU(N) interaction takes the following form: $V^{gg}_p(\mathbf{R}_{12}) \propto b^3_{gg}\mathcal{P}_+$, $V^{ee}_p(\mathbf{R}_{12}) \propto b^3_{ee}\mathcal{P}_+$, $V^{eg}_p(\mathbf{R}_{12}) \propto [(b^+_{eg})^3 + (b^-_{eg})^3]\mathcal{I}/2 + [(b^+_{eg})^3 - (b^-_{eg})^3]\mathcal{P}_{12}/2$. This leads to

the p-wave interaction Hamiltonian in the second quantized form,

$$
\begin{aligned}
H_p = &\frac{3\pi\hbar^2 b_{gg}^3}{2M}\sum_{mm'}\int \mathrm{d}^3\mathbf{R}\,[(\nabla\psi_{gm}^\dagger)\psi_{gm'}^\dagger - \psi_{gm}^\dagger(\nabla\psi_{gm'}^\dagger)]\cdot[\psi_{gm'}(\nabla\psi_{gm}) - (\nabla\psi_{gm'})\psi_{gm}] \\
&+ \frac{3\pi\hbar^2 b_{ee}^3}{2M}\sum_{mm'}\int \mathrm{d}^3\mathbf{R}\,[(\nabla\psi_{em}^\dagger)\psi_{em'}^\dagger - \psi_{em}^\dagger(\nabla\psi_{em'}^\dagger)]\cdot[\psi_{em'}(\nabla\psi_{em}) - (\nabla\psi_{em'})\psi_{em}] \\
&+ \frac{3\pi\hbar^2[(b_{eg}^+)^3 + (b_{eg}^-)^3]}{2M}\sum_{mm'}\int \mathrm{d}^3\mathbf{R}\,[(\nabla\psi_{gm}^\dagger)\psi_{em'}^\dagger - \psi_{gm}^\dagger(\nabla\psi_{em'}^\dagger)]\cdot[\psi_{em'}(\nabla\psi_{gm}) - (\nabla\psi_{em'})\psi_{gm}] \\
&+ \frac{3\pi\hbar^2[(b_{eg}^+)^3 - (b_{eg}^-)^3]}{2M}\sum_{mm'}\int \mathrm{d}^3\mathbf{R}\,[(\nabla\psi_{gm}^\dagger)\psi_{em'}^\dagger - \psi_{gm}^\dagger(\nabla\psi_{em'}^\dagger)]\cdot[\psi_{em}(\nabla\psi_{gm'}) - (\nabla\psi_{em})\psi_{gm'}].
\end{aligned}
\tag{S2}
$$

We focus on the least magnetically sensitive clock transition in ^{87}Sr, $|^1S_0, m_F = \pm 5/2\rangle \rightarrow |^3P_0, m_F = \pm 3/2\rangle$, denoted by $|\tilde{g}\rangle$ and $|\tilde{e}\rangle$ respectively. In a large magnetic field, the flip-flop process of nuclear spin states in Eq. (S1) and Eq. (S2) can be ignored, so the interaction Hamiltonian including s-wave and p-wave contributions can be restricted to these two states, so

$$
\begin{aligned}
H_{\mathrm{int}} = &\frac{2\pi\hbar^2(a_{eg}^- + a_{eg}^+)}{M}\int \mathrm{d}^3\mathbf{R}\,\psi_{\tilde{e}}^\dagger(\mathbf{R})\psi_{\tilde{g}}^\dagger(\mathbf{R})\psi_{\tilde{g}}(\mathbf{R})\psi_{\tilde{e}}(\mathbf{R}) \\
&+ \frac{3\pi\hbar^2 b_{gg}^3}{2M}\int \mathrm{d}^3\mathbf{R}\,[(\nabla\psi_{\tilde{g}}^\dagger)\psi_{\tilde{g}}^\dagger - \psi_{\tilde{g}}^\dagger(\nabla\psi_{\tilde{g}}^\dagger)]\cdot[\psi_{\tilde{g}}(\nabla\psi_{\tilde{g}}) - (\nabla\psi_{\tilde{g}})\psi_{\tilde{g}}] \\
&+ \frac{3\pi\hbar^2 b_{ee}^3}{2M}\int \mathrm{d}^3\mathbf{R}\,[(\nabla\psi_{\tilde{e}}^\dagger)\psi_{\tilde{e}}^\dagger - \psi_{\tilde{e}}^\dagger(\nabla\psi_{\tilde{e}}^\dagger)]\cdot[\psi_{\tilde{e}}(\nabla\psi_{\tilde{e}}) - (\nabla\psi_{\tilde{e}})\psi_{\tilde{e}}] \\
&+ \frac{3\pi\hbar^2[(b_{eg}^+)^3 + (b_{eg}^-)^3]}{2M}\int \mathrm{d}^3\mathbf{R}\,[(\nabla\psi_{\tilde{g}}^\dagger)\psi_{\tilde{e}}^\dagger - \psi_{\tilde{g}}^\dagger(\nabla\psi_{\tilde{e}}^\dagger)]\cdot[\psi_{\tilde{e}}(\nabla\psi_{\tilde{g}}) - (\nabla\psi_{\tilde{e}})\psi_{\tilde{g}}].
\end{aligned}
\tag{S3}
$$

S1.2 Spin model for the carrier transition

As described in the main text, our experimental system is a vertical 1D lattice with magic wavelength ($\lambda_L = 813$ nm), so the external trapping potential $V_{\mathrm{ext}}(\mathbf{R})$ is the same for $|\tilde{g}\rangle$ and $|\tilde{e}\rangle$ states. To the leading order, we have

$$
V_{\mathrm{ext}}(\mathbf{R}) \approx V_0\sin^2(k_L Z) + MgZ + \frac{1}{2}M\omega_R^2(X^2 + Y^2). \tag{S4}
$$

Here, $k_L = 2\pi/\lambda_L$ is the wavenumber of the lattice that sets the atomic recoil energy $E_{rec} = \hbar^2 k_L^2/2M$, g is the gravitational acceleration, and ω_R is the radial trapping frequency. In addition, the clock laser ($\lambda_c = 698$ nm), aligned with the lattice direction, drives the transitions between $|\tilde{g}\rangle$ and $|\tilde{e}\rangle$ states with bare Rabi frequency Ω and detuning δ. In the rotating frame of the clock laser, the second quantized Hamiltonian take the following form,

$$H = H_0 + H_{\text{int}} + H_{\text{laser}}, \tag{S5}$$

where

$$H_0 = \sum_{\alpha=\{\tilde{g},\tilde{e}\}} \int \mathrm{d}^3\mathbf{R}\, \psi_\alpha^\dagger(\mathbf{R}) \Big[-\frac{\hbar^2}{2M}\nabla^2 + V_{\text{ext}}(\mathbf{R}) \Big] \psi_\alpha(\mathbf{R}), \tag{S6}$$

H_{int} is given by Eq. (S3), and

$$H_{\text{laser}} = \frac{\hbar\Omega}{2} \int \mathrm{d}^3\mathbf{R} \Big[\mathrm{e}^{ik_c Z} \psi_{\tilde{e}}^\dagger(\mathbf{R}) \psi_{\tilde{g}}(\mathbf{R}) + \text{h.c.} \Big] - \frac{\hbar\delta}{2} \int \mathrm{d}^3\mathbf{R} \Big[\psi_{\tilde{e}}^\dagger(\mathbf{R}) \psi_{\tilde{e}}(\mathbf{R}) - \psi_{\tilde{g}}^\dagger(\mathbf{R}) \psi_{\tilde{g}}(\mathbf{R}) \Big]. \tag{S7}$$

Here, $k_c = 2\pi/\lambda_c$ is the wavenumber of the clock laser, and $\psi_\alpha(\mathbf{R})$ is the annihilation field operator for a fermionic atom of internal state α.

Our experiment operates in the regime where the collisional rate of relaxation for motional degrees of freedom is slower than internal spin dynamics and trapping frequencies (*28, 29, 36–38*). This condition ensures only internal levels evolve while atoms remain frozen in their single-particle eigenstates during the dynamics. We first focus on the case of the carrier transition, where the clock laser couples the following two single particle states: $\mathbf{n}$, $|\uparrow_\mathbf{n}\rangle \equiv |\tilde{e}; n_X, n_Y, W_n\rangle$ and $|\downarrow_\mathbf{n}\rangle \equiv |\tilde{g}; n_X, n_Y, W_n\rangle$, where $\mathbf{n} = \{n_X, n_Y, n\}$, with n_X, n_Y denoting the radial harmonic oscillator modes and n the lattice site index of the center of the Wannier-Stark state $|W_n\rangle$. We expand the field operator $\psi_\alpha(\mathbf{R})$ in terms of single-particle eigenstates as follows,

$$\psi_{\tilde{e}}(\mathbf{R}) = \sum_\mathbf{n} \phi_{n_X}(X)\phi_{n_Y}(Y)W_n(Z)c_{\mathbf{n}\uparrow}, \quad \psi_{\tilde{g}}(\mathbf{R}) = \sum_\mathbf{n} \phi_{n_X}(X)\phi_{n_Y}(Y)W_n(Z)c_{\mathbf{n}\downarrow}, \tag{S8}$$

where $c_{\mathbf{n}\uparrow}$ and $c_{\mathbf{n}\downarrow}$ are fermionic annihilation operators for $|\uparrow_{\mathbf{n}}\rangle$ and $|\downarrow_{\mathbf{n}}\rangle$ states respectively. Here, the harmonic oscillator wave function is

$$\phi_{n_X}(X) = \frac{1}{\sqrt{2^{n_X} n_X!}} \left(\frac{M\omega_R}{\pi\hbar}\right)^{1/4} \mathrm{e}^{-M\omega_R X^2/2\hbar} H_{n_X}\left(\sqrt{\frac{M\omega_r}{\hbar}} X\right), \tag{S9}$$

where $H_{n_X}(X)$ are Hermite polynomials. The wave function for the Wannier-Stark state is

$$W_n(Z) = \sum_m \mathcal{J}_{m-n}\left(\frac{2J_0}{Mga_l}\right) w(Z - ma_L), \tag{S10}$$

where $\mathcal{J}_n(x)$ are Bessel functions, $J_0 \approx (4/\sqrt{\pi}) E_{rec}^{1/4} V_0^{3/4} \exp[-2\sqrt{V_0/E_{rec}}]$ is the ground band nearest-neighbor tunneling energy, $a_L = \lambda_L/2$ is the lattice spacing, and $w(Z)$ is the ground band Wannier function centering at $Z = 0$.

Under the frozen mode approximation, we treat each atom as a spin-1/2 system spanned by $|\uparrow_{\mathbf{n}}\rangle$ and $|\downarrow_{\mathbf{n}}\rangle$ states. Therefore, we define the spin operators,

$$\begin{aligned} S^x_{\mathbf{n}} &= \frac{1}{2}(c^\dagger_{\mathbf{n}\uparrow} c_{\mathbf{n}\downarrow} + c^\dagger_{\mathbf{n}\downarrow} c_{\mathbf{n}\uparrow}), \quad S^y_{\mathbf{n}} = -\frac{i}{2}(c^\dagger_{\mathbf{n}\uparrow} c_{\mathbf{n}\downarrow} - c^\dagger_{\mathbf{n}\downarrow} c_{\mathbf{n}\uparrow}), \\ S^z_{\mathbf{n}} &= \frac{1}{2}(c^\dagger_{\mathbf{n}\uparrow} c_{\mathbf{n}\uparrow} - c^\dagger_{\mathbf{n}\downarrow} c_{\mathbf{n}\downarrow}), \quad N_{\mathbf{n}} = c^\dagger_{\mathbf{n}\uparrow} c_{\mathbf{n}\uparrow} + c^\dagger_{\mathbf{n}\downarrow} c_{\mathbf{n}\downarrow}, \end{aligned} \tag{S11}$$

and rewrite the interaction Hamiltonian,

$$H_{\mathrm{int}}/\hbar = \sum_{\substack{\mathbf{nm} \\ (\mathbf{n}\neq\mathbf{m})}} \left[J^\perp_{\mathbf{nm}} \mathbf{S}_{\mathbf{n}} \cdot \mathbf{S}_{\mathbf{m}} + \chi_{\mathbf{nm}} S^z_{\mathbf{n}} S^z_{\mathbf{m}} + \frac{C_{\mathbf{nm}}}{2}(S^z_{\mathbf{n}} N_{\mathbf{m}} + N_{\mathbf{n}} S^z_{\mathbf{m}}) \right], \tag{S12}$$

where

$$\begin{aligned} J^\perp_{\mathbf{nm}} &= \eta_{|n-m|}(V^{eg}_{\mathbf{nm}} - U^{eg}_{\mathbf{nm}})/2, \quad \chi_{\mathbf{nm}} = \eta_{|n-m|}(V^{ee}_{\mathbf{nm}} + V^{gg}_{\mathbf{nm}} - 2V^{eg}_{\mathbf{nm}})/2, \\ C_{\mathbf{nm}} &= \eta_{|n-m|}(V^{ee}_{\mathbf{nm}} - V^{gg}_{\mathbf{nm}})/2. \end{aligned} \tag{S13}$$

Here, $\eta_{|n-m|}$ is a dimensionless overlap integral of Wannier-Stark states defined as

$$\eta_{|n-m|} = \frac{\lambda_L}{\sqrt{2\pi}} \left(\frac{V_0}{E_{rec}}\right)^{-1/4} \int \mathrm{d}Z\, [W_n(Z)]^2 [W_m(Z)]^2. \tag{S14}$$

$U^{\alpha\beta}_{\mathbf{nm}}$ and $V^{\alpha\beta}_{\mathbf{nm}}$ are s-wave and p-wave interaction parameters respectively ($\alpha, \beta = \{g, e\}$),

$$U^{\alpha\beta}_{\mathbf{nm}} = \frac{8\pi\hbar a_{\alpha\beta}}{M} s_{n_x m_x} s_{n_y m_y} \frac{k_L}{\sqrt{2\pi}} \left(\frac{V_0}{E_{rec}}\right)^{1/4},$$
$$V^{\alpha\beta}_{\mathbf{nm}} = \frac{6\pi\hbar b^3_{\alpha\beta}}{M} (p_{n_x m_x} s_{n_y m_y} + s_{n_x m_x} p_{n_y m_y}) \frac{k_L}{\sqrt{2\pi}} \left(\frac{V_0}{E_{rec}}\right)^{1/4}, \tag{S15}$$

where $a_{eg} \equiv (a^+_{eg} + a^-_{eg})/2$, $b^3_{eg} \equiv [(b^+_{eg})^3 + (b^-_{eg})^3]/2$, $s_{nm} = \int \mathrm{d}X\, [\phi_n(X)]^2[\phi_m(X)]^2$, and $p_{nm} = \int \mathrm{d}X\, [(\partial_X \phi_n(X))\phi_m(X) - \phi_n(X)(\partial_X \phi_m(X))]^2$. Note that in $V^{\alpha\beta}_{\mathbf{nm}}$ we ignore the p-wave contributions in the $\hat{Z}$ direction, because its leading order terms are overlap matrix elements of gradients of Wannier functions in nearest-neighbor lattice sites based on the expansion in Eq. (S10). These matrix elements are small for parameters used in the experiment.

On the carrier transition, H_{laser} becomes

$$H_{\text{laser}}/\hbar = \frac{1}{2}\sum_{\mathbf{n}} (\Omega_{\mathbf{n}} S^+_{\mathbf{n}} + \text{h.c.}) - \delta \sum_{\mathbf{n}} S^z_{\mathbf{n}}, \tag{S16}$$

where

$$\Omega_{\mathbf{n}} = \Omega \int \mathrm{d}Z\, \mathrm{e}^{ik_c Z} [W_n(Z)]^2 = \Omega_0 \mathrm{e}^{in\varphi}. \tag{S17}$$

Here, $\varphi = k_c a_L = \pi\lambda_L/\lambda_c$ is the clock laser phase difference between nearest-neighbor Wannier-Stark states, generating spin-orbit coupling. The Rabi frequency for carrier transition is

$$\Omega_0 = \Omega \cdot \mathcal{C} \mathcal{J}_0\left(\frac{4J_0}{Mga_L} \sin(\varphi/2)\right), \tag{S18}$$

where $\mathcal{C} = \int \mathrm{d}Z\, \mathrm{e}^{ik_c Z}[w(Z)]^2 \approx \exp\left[-\lambda_L^2/4\lambda_c^2\sqrt{V_0/E_{rec}}\right]$. The dependence of Ω_0 on lattice depth V_0 is shown in Fig. S1(A). In the following discussions, it is convenient to remove the phase dependence on lattice sites in the H_{laser} term by a gauge transformation $\tilde{c}_{\mathbf{n}\uparrow} = \mathrm{e}^{-in\varphi} c_{\mathbf{n}\uparrow}$, $\tilde{c}_{\mathbf{n}\downarrow} = c_{\mathbf{n}\downarrow}$. Under the gauge transformation the spin operators become:

$$\tilde{S}^x_{\mathbf{n}} = \cos(n\varphi) S^x_{\mathbf{n}} - \sin(n\varphi) S^y_{\mathbf{n}}, \quad \tilde{S}^y_{\mathbf{n}} = \sin(n\varphi) S^x_{\mathbf{n}} + \cos(n\varphi) S^y_{\mathbf{n}},$$
$$\tilde{S}^z_{\mathbf{n}} = S^z_{\mathbf{n}}, \quad \tilde{N}_{\mathbf{n}} = N_{\mathbf{n}}. \tag{S19}$$

Combining the discussions above, the effective Hamiltonian in the gauged frame becomes

$$H/\hbar = \sum_{\substack{\mathbf{nm} \\ (\mathbf{n}\neq\mathbf{m})}} \left[\tilde{J}^{\perp}_{\mathbf{nm}} \tilde{\mathbf{S}}_{\mathbf{n}} \cdot \tilde{\mathbf{S}}_{\mathbf{m}} + \tilde{\chi}_{\mathbf{nm}} \tilde{S}^z_{\mathbf{n}} \tilde{S}^z_{\mathbf{m}} + D_{\mathbf{nm}} (\tilde{S}^x_{\mathbf{n}} \tilde{S}^y_{\mathbf{m}} - \tilde{S}^y_{\mathbf{n}} \tilde{S}^x_{\mathbf{m}}) + \frac{C_{\mathbf{nm}}}{2} (\tilde{S}^z_{\mathbf{n}} \tilde{N}_{\mathbf{m}} + \tilde{N}_{\mathbf{n}} \tilde{S}^z_{\mathbf{m}})\right] - \hbar\delta \sum_{\mathbf{n}} \tilde{S}^z_{\mathbf{n}} + \hbar\Omega_0 \sum_{\mathbf{n}} \tilde{S}^x_{\mathbf{n}}, \tag{S20}$$

where $\tilde{J}^{\perp}_{\mathbf{nm}} = \cos[(n-m)\varphi] J^{\perp}_{\mathbf{nm}}$, $\tilde{\chi}_{\mathbf{nm}} = \chi_{\mathbf{nm}} + J^{\perp}_{\mathbf{nm}} - \tilde{J}^{\perp}_{\mathbf{nm}}$, and $D_{\mathbf{nm}} = -\sin[(n-m)\varphi] J^{\perp}_{\mathbf{nm}}$.

Now we discuss the dependence of interaction parameters $\tilde{J}^{\perp}_{\mathbf{nm}}$, $\tilde{\chi}_{\mathbf{nm}}$ and $D_{\mathbf{nm}}$ on radial harmonic oscillator modes (n_X, n_Y, m_X, m_Y) and the distance along lattice direction $(|n-m|)$. As reported in (*28, 36, 37*), the overlap integrals are not overly sensitive to the radial modes in consideration, allowing us to simplify the Hamiltonian dynamics in terms of collective spin operators at each lattice site, $S^{x,y,z}_n = \sum_{n_X n_Y} \tilde{S}^{x,y,z}_{\mathbf{n}}$, $N_n = \sum_{n_X n_Y} \tilde{N}_{\mathbf{n}}$. Due to the partial delocalization of the Wannier-Stark states along the lattice direction, the dominant terms are on-site and nearest-neighbor interactions. Since the characteristic s-wave interaction strength is much larger than p-wave interaction strength at ultracold temperatures (~ 100 nK in our case), we include p-wave interaction only for on-site terms. All these approximations simplify Eq. (S20) into a large-spin Hamiltonian in a 1D lattice,

$$\begin{aligned} H &= H_{\text{on-site}} + H_{\text{off-site}} + H_{\text{laser}}, \\ H_{\text{on-site}}/\hbar &= \sum_n \left[J^{\perp}_0 \mathbf{S}_n \cdot \mathbf{S}_n + \chi_0 S^z_n S^z_n + C_0 N_n S^z_n\right], \\ H_{\text{off-site}}/\hbar &= \sum_n \left[J^{\perp}_1 \mathbf{S}_n \cdot \mathbf{S}_{n+1} + \chi_1 S^z_n S^z_{n+1} + D_1 (S^x_n S^y_{n+1} - S^y_n S^x_{n+1})\right], \\ H_{\text{laser}}/\hbar &= \sum_n \left[-\delta S^z_n + \Omega_0 S^x_n\right]. \end{aligned} \tag{S21}$$

The interaction parameters for these collective spin operators are calculated by performing a thermal average over radial harmonic oscillator modes,

$$\begin{aligned} J^{\perp}_0 &= \eta_0 (V_{eg} - U_{eg})/2, \quad \chi_0 = \eta_0 (V_{ee} + V_{gg} - 2V_{eg})/2, \quad C_0 = \eta_0 (V_{ee} - V_{gg})/2, \\ J^{\perp}_1 &= -\eta_1 U_{eg} \cos\varphi, \quad \chi_1 = -\eta_1 U_{eg} (1 - \cos\varphi), \quad D_1 = -\eta_1 U_{eg} \sin\varphi. \end{aligned} \tag{S22}$$

Here, η_0 and η_1 are dimensionless overlap integrals for on-site and nearest-neighbor interaction respectively [defined in Eq. (S14)], and the thermal average for s-wave ($U_{\alpha\beta}$) and p-wave ($V_{\alpha\beta}$) interaction strengths are

$$U_{\alpha\beta} = \frac{8\pi\hbar a_{\alpha\beta}}{M}\frac{M\omega_R^2}{4\pi k_B T}\frac{k_L}{\sqrt{2\pi}}\left(\frac{V_0}{E_{rec}}\right)^{1/4}, \quad V_{\alpha\beta} = \frac{6\pi\hbar b_{\alpha\beta}^3}{M}\frac{1}{\pi}\left(\frac{M\omega_R}{\hbar}\right)^2\frac{k_L}{\sqrt{2\pi}}\left(\frac{V_0}{E_{rec}}\right)^{1/4}. \tag{S23}$$

The dependence of interaction parameters χ_0, χ_1, C_0 on lattice depth V_0 is shown in Fig. S1B.

S1.3 Density shift of the carrier transition

As described in the main text, we measure the density shift of the carrier transition in Rabi spectroscopy. Note that the clock transition frequency is obtained by the average of two frequencies with the same excitation fraction on the positive and negative detuned side of the π-pulse Rabi spectrum, typically with an excitation fraction near 0.45 (the maximum excitation fraction is near 0.9). The density shift per atom

$$\Delta\nu = \frac{\delta_{\text{left}} + \delta_{\text{right}}}{4\pi N_{\text{loc}}}, \tag{S24}$$

where δ_{left} and δ_{right} are the laser detuning from clock transition resonance for the excitation fraction we set on the positive and negative detuned side of the Rabi spectrum, and $N_{\text{loc}} = \frac{1}{2L+1}\sum_{m=-L}^{L} N_{n+m}$ is the averaged atom number per site in a local region centered around site n. The local region is $2L + 1 \sim 15$ lattice sites, corresponding to our 6 μm imaging resolution.

To calculate the density shift, we apply a mean-field approximation to Eq. (S21),

$$H_{\text{MF}}/\hbar = \sum_n \mathbf{S}_n \cdot \mathbf{B}_n, \tag{S25}$$

where

$$
\begin{aligned}
B_n^x &= \Omega_0 + J_1^\perp(\langle S_{n-1}^x\rangle + \langle S_{n+1}^x\rangle) + D_1(\langle S_{n+1}^y\rangle - \langle S_{n-1}^y\rangle),\\
B_n^y &= J_1^\perp(\langle S_{n-1}^y\rangle + \langle S_{n+1}^y\rangle) - D_1(\langle S_{n+1}^x\rangle - \langle S_{n-1}^x\rangle),\\
B_n^z &= -\delta + 2\chi_0\langle S_n^z\rangle + C_0 N_n + J_1^\perp(\langle S_{n-1}^z\rangle + \langle S_{n+1}^z\rangle) + \chi_1(\langle S_{n-1}^z\rangle + \langle S_{n+1}^z\rangle).
\end{aligned}
\tag{S26}
$$

Note that we drop the $J_0^\perp$ term in Eq. (S21) because this term is a constant for any collective state at each lattice site.

We further simplify Eq. (S25) by assuming all lattice sites share the same atom number $N_{\rm loc}$ in a local region (15 lattice sites). We calculate the spin dynamics in this local region by assuming translationally invariant conditions $\langle S_n^{x,y,z}\rangle = \langle S^{x,y,z}\rangle$ to Eq. (S26), where $\langle S^{x,y,z}\rangle = \frac{1}{2L+1}\sum_{m=-L}^{L}\langle S_{n+m}^{x,y,z}\rangle$. In this way, we have a homogeneous field on each site, $\mathbf{B}_n = \mathbf{B}$. The mean-field Hamiltonian becomes

$$H_{\rm MF}/\hbar = \sum_n \mathbf{S}_n \cdot \mathbf{B}^\perp, \tag{S27}$$

where $\mathbf{B}^\perp \perp \langle\mathbf{S}\rangle$ is the perpendicular component of $\mathbf{B}$, with

$$\mathbf{B}^\perp = \{\Omega_0, 0, -\delta + 2(\chi_0+\chi_1)\langle S^z\rangle + C_0 N_{\rm loc}\}. \tag{S28}$$

Dropping the parallel component of $\mathbf{B}$ because it does not contribute to the mean-field dynamics,

$$\frac{\mathrm{d}}{\mathrm{d}t}\langle\mathbf{S}_n\rangle = \mathbf{B}^\perp \times \langle\mathbf{S}_n\rangle. \tag{S29}$$

Using the mean-field equations above, we simulate the experimental protocol and obtain theoretical predictions for the density shift. In Rabi spectroscopy, we initialize all the atoms in the ground state ($\langle S_n^z\rangle = -N_{\rm loc}/2$) for $g \to e$ case, and all the atoms in the excited state ($\langle S_n^z\rangle = N_{\rm loc}/2$) for $e \to g$ case.

From Eq. (S28), we obtain a simple expression for the density shift by setting it to be

the value of δ at which $\mathbf{B}_z^{\perp} = 0$:

$$\begin{aligned} \Delta\nu_{\alpha\to\beta} &= \Delta\nu^s_{\alpha\to\beta} + \Delta\nu^p_{\alpha\to\beta}, \\ 2\pi\Delta\nu^p_{\alpha\to\beta} \approx 2\chi_0\varsigma^z_{\alpha\to\beta} + C_0, &\qquad 2\pi\Delta\nu^s_{\alpha\to\beta} \approx 2\chi_1\varsigma^z_{\alpha\to\beta}. \end{aligned} \tag{S30}$$

Here, $\Delta\nu^{s,p}_{\alpha\to\beta}$ are the s-wave and p-wave contributions to the density shift, $\varsigma^z_{\alpha\to\beta}$ is a fitting parameter that accounts for the time evolution of $\langle S^z\rangle/N_{\rm loc}$ during the Rabi dynamics, which depends on the details of the Rabi drive such as the pulse area, excitation fraction, and initial conditions used in the experiment, $g\to e$ or $e\to g$. Based on our experimental condition in the carrier transition, we find $\varsigma^z_{g\to e} = -0.12$ and $\varsigma^z_{e\to g} = 0.095$ [see Fig. S1(C)]. Note that $\Delta\nu^p_{\alpha\to\beta}$ are generated by on-site p-wave interactions, while $\Delta\nu^s_{\alpha\to\beta}$ are generated by nearest-neighbor s-wave interaction. This dependence allows us to control the density shift by adjusting the spatial extension of the Wannier-Stark states, which is tunable by varying the lattice depth, the key idea to eliminating the density shift presented in the main text.

S1.4 Spin model for off-site Wannier-Stark transitions

Apart from the carrier transition, we can also drive transitions to other Wannier-Stark states by using the clock laser to couple two different internal and motional states of an atom, $|\uparrow_{\mathbf{n}}\rangle \equiv |\tilde{e}; n_X, n_Y, W_{n+l}\rangle$ and $|\downarrow_{\mathbf{n}}\rangle \equiv |\tilde{g}; n_X, n_Y, W_n\rangle$, with $l = \pm1, \pm2, \cdots$. Compared to the carrier transition, the subscript $\mathbf{n}$ labels different motional states for $|\uparrow_{\mathbf{n}}\rangle$ and $|\downarrow_{\mathbf{n}}\rangle$ states in off-site Wannier-Stark transitions. In this case, we expand the field operator $\psi_\alpha(\mathbf{r})$ in terms of single-particle eigenstates,

$$\psi_{\tilde{e}}(\mathbf{R}) = \sum_{\mathbf{n}} \phi_{n_X}(X)\phi_{n_Y}(Y)W_{n+l}(Z)c_{\mathbf{n}\uparrow}, \quad \psi_{\tilde{g}}(\mathbf{R}) = \sum_{\mathbf{n}} \phi_{n_X}(X)\phi_{n_Y}(Y)W_n(Z)c_{\mathbf{n}\downarrow}. \tag{S31}$$

Similar to the frozen-mode approximation used for the carrier transition, we treat each atom as a spin-1/2 system spanned by the $|\uparrow_{\mathbf{n}}\rangle$ and $|\downarrow_{\mathbf{n}}\rangle$ states defined for the specific

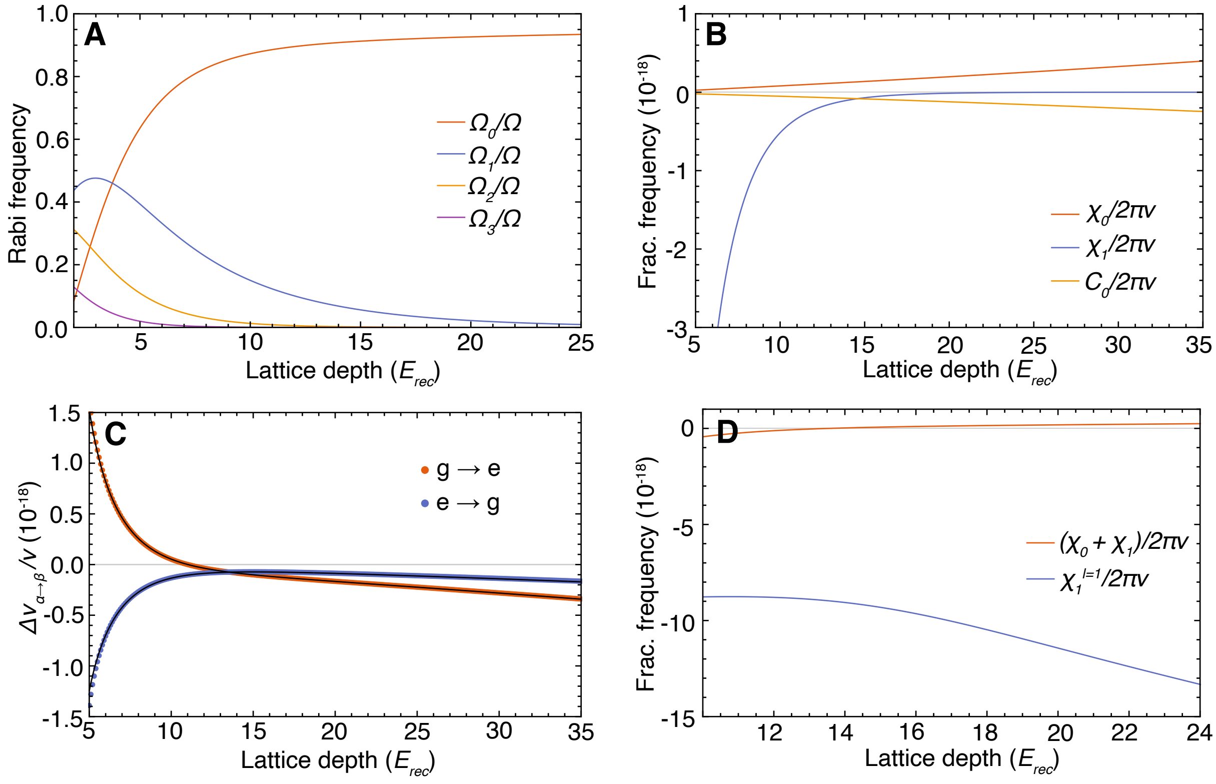

Figure S1: **Spin Model Parameters.** (**A**) Rabi frequency for the carrier transition (Ω_0) and $l = 1, 2, 3$ Wannier-Stark sidebands ($\Omega_1, \Omega_2, \Omega_3$) as a function of lattice depth. (**B**) Spin model parameters for the carrier transition as a function of lattice depth. The radial temperature at each lattice depth used is the reported experimental value (See Fig. S4. (**C**) Theoretical predictions of the fractional frequency shift per atom (orange points for $g \to e$ case, blue points for $e \to g$ case) and numerical fits based on Eq. (S30) shown as black lines. The fitting parameter used are $\varsigma^z_{g\to e} = -0.12$ and $\varsigma^z_{e\to g} = 0.095$. (**D**) Wannier-Stark sideband interaction parameter ($\chi_1^{l=1}$) compared to the carrier transition parameter ($\chi_0 + \chi_1$), with the former significantly enhanced compared to the latter. In this case as well the radial temperature at each lattice depth is the reported experimental value S2.1.

Wannier-Stark states coupled by the laser, and rewrite the interaction Hamiltonian in terms of the corresponding spin operators,

$$H_{\text{int}}/\hbar = \sum_{\substack{\mathbf{nm} \\ (\mathbf{n}\neq\mathbf{m})}} \left[J^{\perp,l}_{\mathbf{nm}} \mathbf{S}_{\mathbf{n}} \cdot \mathbf{S}_{\mathbf{m}} + \chi^{l}_{\mathbf{nm}} S^z_{\mathbf{n}} S^z_{\mathbf{m}} + \frac{C_{\mathbf{nm}}}{2}(S^z_{\mathbf{n}} N_{\mathbf{m}} + N_{\mathbf{n}} S^z_{\mathbf{m}}) + \frac{K^l_{\mathbf{nm}}}{2}(S^z_{\mathbf{n}} N_{\mathbf{m}} - N_{\mathbf{n}} S^z_{\mathbf{m}}) \right], \tag{S32}$$

where

$$\begin{aligned} J^{\perp,l}_{\mathbf{nm}} &= \eta^{\text{ex},l}_{|n-m|}(V^{\text{eg}}_{\mathbf{nm}} - U^{\text{eg}}_{\mathbf{nm}})/2, \\ \chi^l_{\mathbf{nm}} &= \eta_{|n-m|}(V^{ee}_{\mathbf{nm}} + V^{gg}_{\mathbf{nm}})/2 - \eta^{\text{dir},l}_{|n-m|}(V^{eg}_{\mathbf{nm}} + U^{eg}_{\mathbf{nm}})/2 - \eta^{\text{ex},l}_{|n-m|}(V^{\text{eg}}_{\mathbf{nm}} - U^{\text{eg}}_{\mathbf{nm}})/2, \\ C_{\mathbf{nm}} &= \eta_{|n-m|}(V^{ee}_{\mathbf{nm}} - V^{gg}_{\mathbf{nm}})/2, \\ K^l_{\mathbf{nm}} &= \eta^{\text{diff},l}_{nm}(V^{eg}_{\mathbf{nm}} + U^{eg}_{\mathbf{nm}})/2. \end{aligned} \tag{S33}$$

Here, $\eta_{|n-m|}$, $U^{\alpha\beta}_{\mathbf{nm}}$, $V^{\alpha\beta}_{\mathbf{nm}}$ have the same definition as the ones used for the carrier transition [see Eq. (S14) and Eq. (S15)], and the definitions for the extra dimensionless overlap intergrals are

$$\begin{aligned} \eta^{\text{dir},l}_{|n-m|} &= \frac{1}{2}(\eta_{|n-m+l|} + \eta_{|n-m-l|}), \\ \eta^{\text{diff},l}_{nm} &= \frac{1}{2}(\eta_{|n-m+l|} - \eta_{|n-m-l|}), \\ \eta^{\text{ex},l}_{|n-m|} &= \frac{\lambda_L}{\sqrt{2\pi}} \left(\frac{V_0}{E_{rec}}\right)^{-1/4} \int \mathrm{d}Z\, W_n(Z) W_m(Z) W_{n+l}(Z) W_{m+l}(Z). \end{aligned} \tag{S34}$$

Note that the Rabi frequency for the Wannier-Stark sidebands experiences the same spin-orbit coupling phase as the carrier transition. We use the gauge transformation as in the carrier transition to redefine the spin operators [see Eq. (S19)], and the effective Hamiltonian in the gauged frame becomes

$$\begin{aligned} H/\hbar = \sum_{\substack{\mathbf{nm} \\ (\mathbf{n}\neq\mathbf{m})}} \Bigg[& \tilde{J}^{\perp,l}_{\mathbf{nm}} \tilde{\mathbf{S}}_{\mathbf{n}} \cdot \tilde{\mathbf{S}}_{\mathbf{m}} + \tilde{\chi}^l_{\mathbf{nm}} \tilde{S}^z_{\mathbf{n}} \tilde{S}^z_{\mathbf{m}} + D^l_{\mathbf{nm}}(\tilde{S}^x_{\mathbf{n}} \tilde{S}^y_{\mathbf{m}} - \tilde{S}^y_{\mathbf{n}} \tilde{S}^x_{\mathbf{m}}) + \frac{C_{\mathbf{nm}}}{2}(\tilde{S}^z_{\mathbf{n}} \tilde{N}_{\mathbf{m}} + \tilde{N}_{\mathbf{n}} \tilde{S}^z_{\mathbf{m}}) \\ & \frac{K^l_{\mathbf{nm}}}{2}(\tilde{S}^z_{\mathbf{n}} \tilde{N}_{\mathbf{m}} - \tilde{N}_{\mathbf{n}} \tilde{S}^z_{\mathbf{m}}) \Bigg] - \hbar\delta_l \sum_{\mathbf{n}} \tilde{S}^z_{\mathbf{n}} + \hbar\Omega_l \sum_{\mathbf{n}} \tilde{S}^x_{\mathbf{n}}, \end{aligned} \tag{S35}$$

where $\tilde{J}^{\perp,l}_{\mathbf{nm}} = \cos[(n-m)\varphi]J^{\perp}_{\mathbf{nm}}$, $\tilde{\chi}^{l}_{\mathbf{nm}} = \chi^{l}_{\mathbf{nm}} + J^{\perp,l}_{\mathbf{nm}} - \tilde{J}^{\perp,l}_{\mathbf{nm}}$, $D^{l}_{\mathbf{nm}} = -\sin[(n-m)\varphi]J^{\perp,l}_{\mathbf{nm}}$, $\delta_l = \delta - lMga_L/\hbar$, and

$$\Omega_l = \Omega \cdot \mathcal{C}\mathcal{J}_l\left(\frac{4J_0}{Mga_L}\sin(\varphi/2)\right). \tag{S36}$$

The dependence of Ω_l ($l = 1, 2, 3$) on lattice depth V_0 is shown in Fig. S1(A).

In the following discussions, we focus on the $l = 1$ Wannier-Stark transition. Following the same procedure we used for the carrier transition, we can express the Hamiltonian dynamics in terms of collective spin operators, $S^{x,y,z}_n = \sum_{n_x n_y} \tilde{S}^{x,y,z}_{\mathbf{n}}$, $N_n = \sum_{n_x n_y} \tilde{N}_{\mathbf{n}}$. Recall that for the carrier transition we discussed in previous sections, on-site s-wave interactions only gave rise to a constant term ($J^{\perp}_0$ term) which does not play any role in the mean-field dynamics. The dominant interaction comes from on-site p-wave interactions and nearest-neighbor s-wave interactions. However, in the case of the $l = 1$ site-changing Wannier-Stark transition, a ground state atom in $|W_n\rangle$ acquires a non-zero admixture of the excited state in $|W_{n+1}\rangle$. This component can interact with a ground state atom in $|W_{n+1}\rangle$ via s-wave interactions. Since the on-site s-wave interactions play a significant role in this case, we drop all the interaction terms smaller than such on-site s-wave interactions. We can also drop the $K^{l}_{\mathbf{nm}}$ term due to the uniform atom population for lattice sites in a local regime. These approximations lead to the following large-spin Hamiltonian in a 1D lattice,

$$H/\hbar = \sum_n \left[\chi^{l=1}_1 S^z_n S^z_{n+1} - \delta_1 S^z_n + \Omega_1 S^x_n\right], \tag{S37}$$

where

$$\chi^{l=1}_1 = -\eta_0 U_{eg}/2. \tag{S38}$$

In Fig. S1(D), we compare $\chi^{l=1}_1$ with its counterpart $\chi_0 + \chi_1$ in the carrier transition. It is clear that the interaction is significantly enhanced due to site-changing Wannier-Stark transitions.

S1.5 Dynamical phase transition

In the main text we presented theoretical and experimental results on the ferromagnetic to paramagentic dynamical phase transition (DPT) when we address the $l = 1$ Wannier-Stark transition. Given that Eq. (S37) is a large-spin Hamiltonian, its dynamical phase diagram is well captured by a mean-field approximation. Similar to the carrier transition, we apply the translationally invariant condition $\langle S_n^{x,y,z}\rangle = \langle S^{x,y,z}\rangle$ in a local regime (15 lattice sites), where $\langle S^{x,y,z}\rangle = \frac{1}{2L+1}\sum_{m=-L}^{L}\langle S_{n+m}^{x,y,z}\rangle$. This leads to the following mean-field Hamiltonian,

$$H_{\mathrm{MF}}/\hbar = \sum_n \mathbf{S}_n \cdot \mathbf{B}, \tag{S39}$$

where

$$\mathbf{B} = \{\Omega_1, 0, -\delta_1 + 2\chi_1^{l=1}\langle S^z\rangle\}. \tag{S40}$$

Writing mean-field equations can be written in terms of normalized expectation value of collective spin operators on a single site $s^{x,y,z} = 2\langle S^{x,y,z}\rangle/N_{\mathrm{loc}}$,

$$\begin{aligned} \frac{\mathrm{d}}{\mathrm{d}t}s^x &= -N_{\mathrm{loc}}\chi_1^{l=1}s^z s^y + \delta_1 s^y, \\ \frac{\mathrm{d}}{\mathrm{d}t}s^y &= N_{\mathrm{loc}}\chi_1^{l=1}s^z s^x - \delta_1 s^x - \Omega_1 s^z, \\ \frac{\mathrm{d}}{\mathrm{d}t}s^z &= \Omega_1 s^y. \end{aligned} \tag{S41}$$

Note that Eq. (S41) takes the same form as the mean-field equations obtained in (*35, 36*), which predicted a DPT between ferromagnetic and paramagnetic phases.

In general terms, a DPT is characterized by the existence of a critical point separating phases with distinct dynamical properties in many-body systems after a sudden quench. The analog of thermodynamic order parameters is found in long-time average observables, which have a non-analytic dependence on system parameters. We initialize all the atoms in the $|\downarrow\rangle$ state, the ground state of our model when $\delta_1 \to -\infty$, and then perform a sudden quench of the longitudinal field to its final value δ_1. The DPT is signaled by a sharp

change in behavior of the long-time average excitation fraction $\overline{n_\uparrow} = (\overline{s^z}+1)/2$, where $\overline{s^z} = \lim_{T\to\infty}\frac{1}{T}\int_0^T s^z(t)\mathrm{d}t$. In the dynamical ferromagnetic phase, $\overline{n_\uparrow}\approx 0$ persists even when the final longitudinal field δ_1 is varied. In the dynamical paramagnetic phase, $\overline{n_\uparrow}$ dynamically adjusts itself following the change of final longitudinal field δ_1 [See Fig. S2(C)].

In the following, we analyze the critical points for the DPT in our system based on the procedure described in (*35, 36*). Using energy conservation in H_{MF} for an initial state with $s^z=-1$, $s^x=s^y=0$,

$$\frac{N_{\mathrm{loc}}\chi_1^{l=1}}{2}s^z s^z - \delta_1 s^z + \Omega_1 s_x = \frac{N_{\mathrm{loc}}\chi_1^{l=1}}{2} + \delta_1, \tag{S42}$$

as well as the identity,

$$(s^x)^2 + (s^y)^2 + (s^z)^2 = 1. \tag{S43}$$

In the large-N_{loc} limit, we can eliminate s^x and s^y, and obtain the following differential equation for s^z,

$$\frac{1}{2}\left(\frac{\mathrm{d}}{\mathrm{d}t}s^z\right)^2 + V(s^z) = 0, \tag{S44}$$

where

$$\begin{aligned} V(s^z) = (s^z+1)\Bigg\{&\frac{(N_{\mathrm{loc}}\chi_1^{l=1})^2}{8}(s^z)^3 - \left[\frac{(N_{\mathrm{loc}}\chi_1^{l=1})^2}{8} + \frac{N_{\mathrm{loc}}\chi_1^{l=1}\delta_1}{2}\right](s^z)^2 \\ &+ \left[\frac{\delta_1^2+\Omega_1^2}{2} - \frac{(N_{\mathrm{loc}}\chi_1^{l=1})^2}{8}\right]s^z + \left[\frac{\delta_1^2-\Omega_1^2}{2} + \frac{N_{\mathrm{loc}}\chi_1^{l=1}\delta_1}{2} + \frac{(N_{\mathrm{loc}}\chi_1^{l=1})^2}{8}\right]\Bigg\}. \end{aligned} \tag{S45}$$

Our experimental conditions lie in the parameter regime where $N_{\mathrm{loc}}\chi_1^{l=1} < 0$ with a fixed positive Ω_1.

We interpret Eq. (S44) as the Hamiltonian of a classical particle with position s^z moving in the effective potential $V(s^z)$, which is shown in Fig. S2(A). The condition $V(s^z)=0$ determines the physical turnover points of s^z. Since $V(-1)=0$, $V'(-1) = -1$, $V(1)=2\delta_1^2$, this effective potential has at least two real roots in $[-1,1]$. The

dynamics of

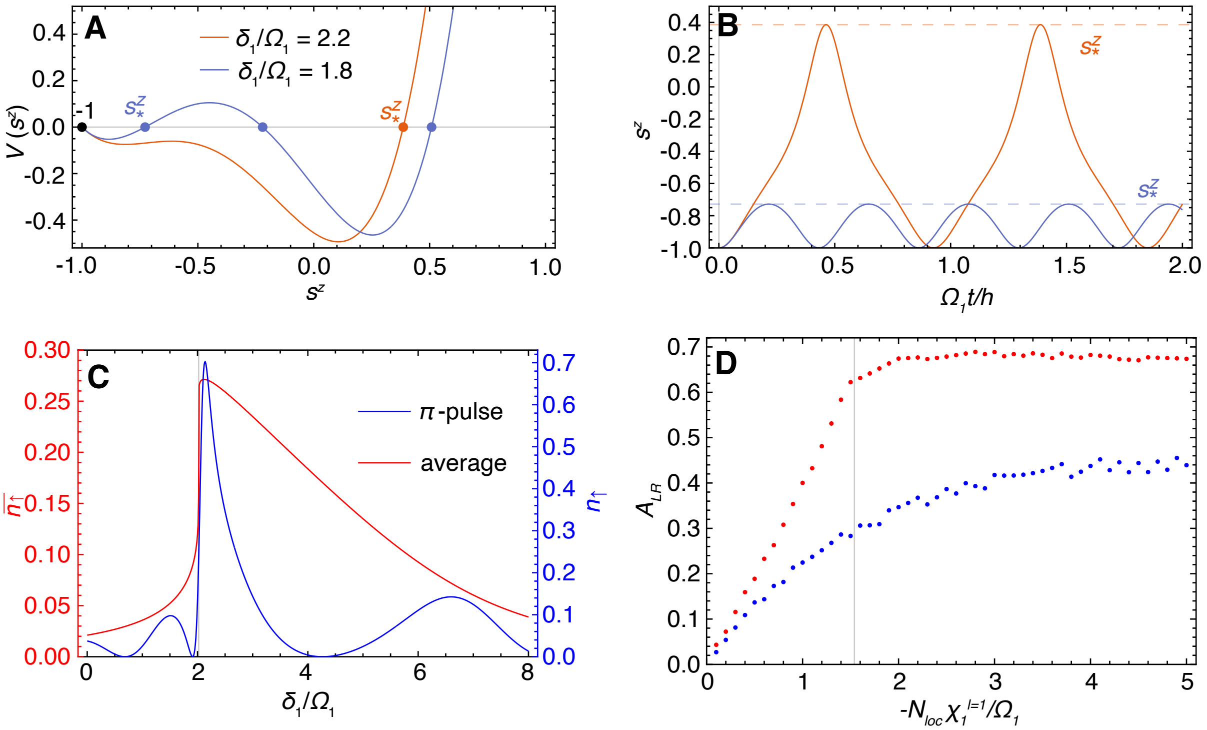

Figure S2: **Dynamical Phase Transition.** (**A**) The effective potential $V(s^z)$ with $N_{\text{loc}}\chi_1^{l=1}/\Omega_1 = -5$. In the case of $\delta_1/\Omega_1 = 2.2$, $V(s^z)$ has two real roots; In the case of $\delta_1/\Omega_1 = 1.8$, $V(s^z)$ has four real roots. The nearest turnover point is labelled by s^z_*, and the jump of s^z_* indicates the DPT. (**B**) The mean-field dynamics of our model with $N_{\text{loc}}\chi_1^{l=1}/\Omega_1 = -5$ and $\delta_1/\Omega_1 = 2.2, 1.8$, which shows a sharp change of mean-field dynamical behavior. The choice of color for the lines is the same as (**A**). (**C**) The long-time average excitation fraction $\overline{n_\uparrow}$ (red line) and the Rabi lineshape after a π-pulse (blue line) with $N_{\text{loc}}\chi_1^{l=1}/\Omega_1 = -5$. The critical point (marked by gray line) that separates the ferromagnetic phase (left) and paramagnetic phase (right) is captured by the maximum derivative in both of the curves. (**D**) Asymmetry of the long-time averaged excitation fraction and Rabi lineshape in (**C**) with the same choice of color. The gray line separates the crossover regime (left) and DPT regime (right).

s^z can be understood as the oscillations between -1 and the nearest turnover point s^z_* [see Fig. S2(B)]. Suppose we start from a $V(s^z)$ with two real roots, and continuously tune the parameters of $V(s^z)$ so that two new real roots appear in between. Then a jump of the nearest turnover point s^z_* should occur in this process. This abrupt change in behavior is what sets the dynamical phase transition [see Fig. S2(A,B)].

To count the number of roots in $V(s^z)$, we factor out the known root $s^z = -1$, and then consider the discriminant $\Delta = 18abcd - 4b^3d + b^2c^2 - 4ac^3 - 27a^2d^2$ of cubic equation $ax^3 + bx^2 + cx + d = 0$. If $\Delta > 0$, the cubic equation has three distinct real roots; if $\Delta < 0$, the cubic equation has one real root. So $\Delta = 0$ sets the critical points of the DPT, presented as the black solid line in Fig. 4(C) of the main text. As shown in Fig. S2(C), the critical points can be captured by the divergence of the first derivative of $\overline{n_\uparrow}$. Similar to (*35, 36*), our experiment measures the excitation fraction at a finite time (after a π pulse) instead of the long-time averaged excitation fraction. Although the derivative does not diverge in experiment, the maximum derivative can still be used to capture the critical point as shown in Fig. S2(C)]. In Fig. 4(C) of the main text, we construct the phase boundary of the DPT with the maximum derivative of the experimental Rabi lineshapes. We find that the many-body decoherence discussed in the next section has negligible effect on the position of the critical points, nevertheless it obscures the sharp features at the DPT expected from Eq. (S39).

Moreover, based on the existence of real roots in equation $\Delta = 0$, we can also differentiate the DPT regime ($N_{\text{loc}}\chi_1^{l=1}/\Omega_1 < -8\sqrt{3}/9$) dominated by interactions and the smooth crossover regime ($-8\sqrt{3}/9 < N_{\text{loc}}\chi_1^{l=1}/\Omega_1 < 0$) dominated by single-particle Rabi flopping where no DPT takes place. Based on our experimental condition ($22E_{rec}$, 190nK and 2.3s π-pulse), the boundary of these two regimes $N_{\text{loc}}\chi_1^{l=1}/\Omega_1 = -8\sqrt{3}/9$ is equivalent to $N_{\text{loc}} = 63.4$, indicated by the black dashed line in Fig. 4(C) of the main text.

These two regimes can also be determined by the asymmetry of the long-time averaged excitation fraction or Rabi lineshape, defined as $A_{LR} = (n_R - n_L)/(n_R + n_L)$. Here, $n_R = \int_{\delta_{max}}^{\delta_{max}+f} n_\uparrow(\delta)\mathrm{d}\delta$, $n_L = \int_{\delta_{max}-f}^{\delta_{max}} n_\uparrow(\delta)\mathrm{d}\delta$, where δ_{max} is the detuning at which the peak value of $n_\uparrow$ is reached, and we choose f to cover almost the entire frequency range of non-vanishing $n_\uparrow$. In Fig. S2(D), we compare the A_{LR} obtained from the long-time averaged excitation fraction and the the one obtained from the Rabi lineshape after a π pulse. In both cases, the asymmetry A_{LR} becomes more pronounced as the atom number increases in the crossover regime, while A_{LR} saturates near the maximum value in the DPT regime. Note that the many-body decoherence discussed in the next section generally reduces the asymmetry. Nevertheless the saturation behavior observed in the DPT regime is maintained. For convenience, in Fig. 4(C) of the main text we normalize the maximum value of A_{LR} obtained from experimental lineshapes to 1.

S2 Additional Experimental Details

S2.1 Sample preparation

Characteristic axial scans at 300 E_{rec} are shown in Fig. S3. Without axial cooling, the red trace, the sample thermally populates many axial modes. Due to the anharmonicity of the trapping potential, transitions between different oscillator levels are resolvable as distinct peaks in both the positively detuned blue and negatively detuned red sidebands. We verify that sideband cooling populates the ground oscillator level by observing the elimination of the red sideband.

The radial temperature T_r over a range of lattice depths is shown in Fig. S4. The piecewise function Eq. 7 is plotted alongside the red experimental data points.

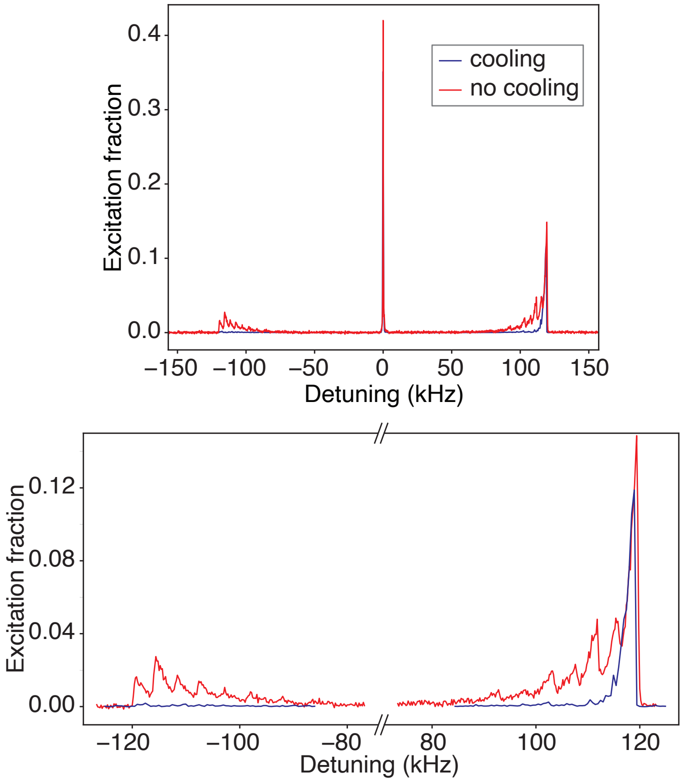

Figure S3: **Sideband Spectroscopy.** Axial sideband spectroscopy with a radially cooled sample at 300 E_{rec}. We scan the detuning of the clock laser from the carrier transition using high intensity clock light to probe the axial mode filling. The red trace was measured without axial sideband cooling. The blue trace was measured after implementing axial sideband cooling, illustrating near perfect sample preparation in the lowest motional band. The different axial transitions are apparent in the axial sidebands enlarged in the lower plot.

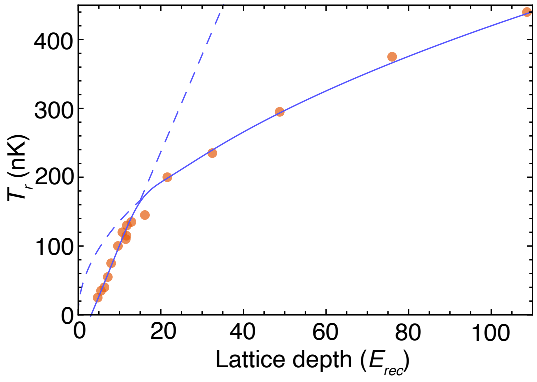

Figure S4: **Radial Temperature.** Radial temperature T_r measured over a range of operational lattice depths V_0 in units of lattice photon recoil energies, E_{rec}. The red points indicate experimental data, and the solid blue lines show the piecewise fit of Eq. 7.

S2.2 Coherence time of off-site Wannier-Stark transitions

We utilize a similar method as in Ref. (*6*) to extract a coherence time on the $|g\,;\,W_n\rangle \rightarrow |e\,;\,W_{n\pm1}\rangle$ transition. Using a Ramsey sequence with a randomly sampled phase for the second pulse, we fit the contrast decay between two spatially resolved regions of the sample as a function of dark time, see Fig. S5.

Due to very strong s-wave interactions on this transition, we observe a significant dependence on local density. While this dependence merits further study, here we operate the system in a relatively low density regime compared to our density shift measurements to determine a near optimal coherence. Fitting a single exponential time decay to the contrast, we measure an atomic coherence time of 20(1) s. The regions of study are selected by fitting a Gaussian to the atomic distribution and selecting two regions from the center to 1.5 times the Gaussian RMS width. Greater coherence time was observed in regions with lower density. Hence what we report here represents the lower bound of atomic coherence for the off-site Wannier-Stark drive. There is no reason to expect its coherence to be less than that of the carrier transition, if one can cleanly separate the

interaction effects.

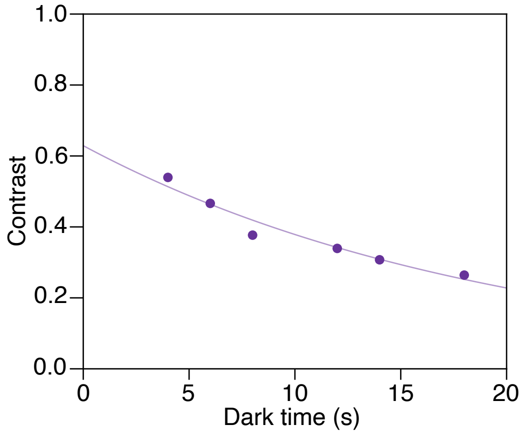

Figure S5: **Coherence Time.** To determine the coherence time of a site-changing Wannier-Stark transition, we use a Ramsey sequence with a randomly sampled phase for the second rotation. As in Ref. (*6*), randomly sampling a phase for a given dark time traces out an ellipse. We fit the ellipse to extract a contrast measurement, reported here as purple dots. The contrast decay is fit with a single exponential with decay time $\tau = 20(1)$ s.

S2.3 Density shift measurement

As described in the main text, we use extended 'clock locks' to measure the spatially dependent average density shift. Fig. S6 illustrates how we measure the density shift coefficients. Using our camera imaging, we construct a density and frequency map of the sample, shown in Fig. S6A and B respectively. Finally, as illustrated in Fig. S6C, we fit this frequency as a function of atom number with a linear function, with the slope the density shift coefficient.

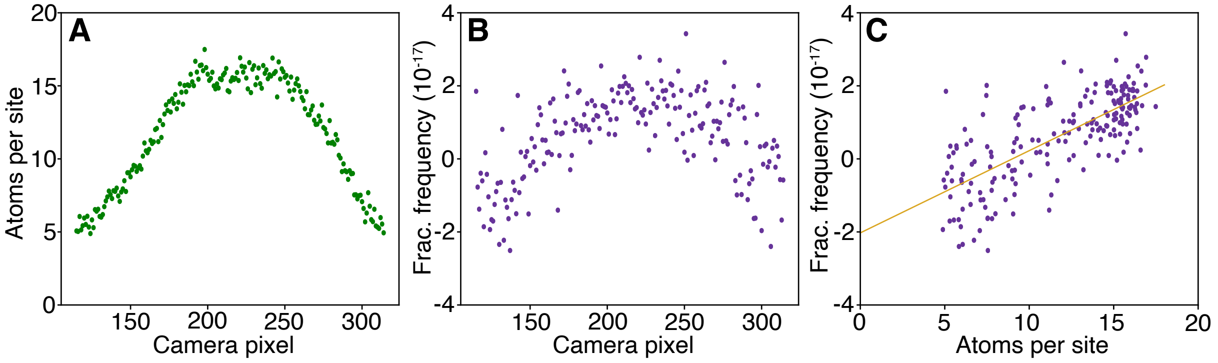

Figure S6: **Measuring the Density Shift.** For each experiment cycle we collect a set of images that allow us to locally determine the excitation fraction and atom number. To optimize the signal, we select a region of interest near the peak density as in Ref. (*6*). With a four point probing scheme, we measure the average atom number at each pixel (**A**) and construct the bare frequency (**B**). The absolute frequency is arbitrary. (**C**) The frequency as a function of atoms per site. The purple points are data and the gold line is a linear fit, the slope of which is the density shift coefficient for this single four point measurement cycle.

S3 Experiment - Theory Comparisons

S3.1 Many-body decoherence in off-site Wannier-Stark transitions

Our theoretical model is based on the frozen-mode approximation, which restricts the accessible Hilbert space of each atom into a spin-1/2 degree of freedom spanned by the $|\uparrow_{\mathbf{n}}\rangle$ and $|\downarrow_{\mathbf{n}}\rangle$ states. Our spin model is valid in the collisionless regime, breaking down at long times or at high enough densities where mode relaxation is not negligible. Since the interaction strength is significantly enhanced when interrogating site-changing WS transitions, as discussed in previous sections, the mode relaxation rate is expected to be more significant. We take into account the mode-changing collisions phenomenologically by adding a density-dependent dephasing term (γ_z) into our mean-field equations for the

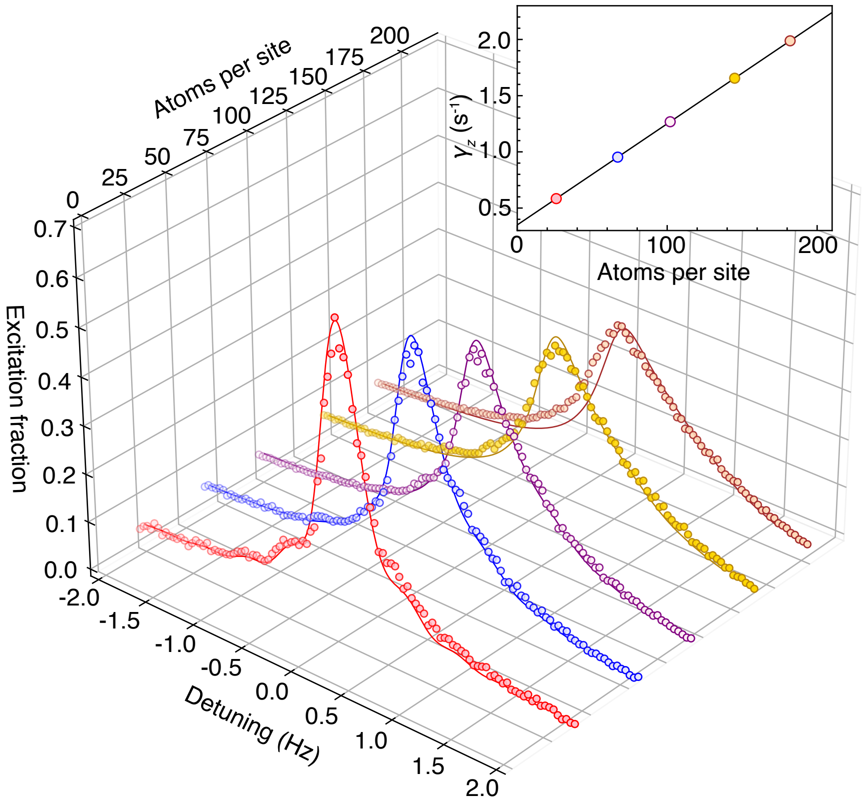

Figure S7: **Many-body Decoherence.** Rabi lineshapes for the $l = 1$ Wannier-Stark $l = 1$ transition and corresponding theoretical fits at different N_{loc}. The dephasing rate γ_z is the only fitting parameter, which is shown in the inset using the same color as the Rabi lineshapes. The linear dependence of γ_z on atom number per site ($\gamma_z = 0.35 + 0.009N_{\text{loc}}$) confirms that the dephasing effect is generated by mode-changing collisions.

$l = 1$ Wannier-Stark sideband [see Eq. (S41)],

$$\begin{aligned}\frac{\mathrm{d}}{\mathrm{d}t}s^x &= -N_{\mathrm{loc}}\chi_1^{l=1}s^z s^y + \delta_1 s^y - \gamma_z s^x,\\ \frac{\mathrm{d}}{\mathrm{d}t}s^y &= N_{\mathrm{loc}}\chi_1^{l=1}s^z s^x - \delta_1 s^x - \Omega_1 s^z - \gamma_z s^y,\\ \frac{\mathrm{d}}{\mathrm{d}t}s^z &= \Omega_1 s^y,\end{aligned} \tag{S46}$$

We use γ_z as a fitting parameter and find it has a linear dependence on N_{loc} as expected from mode changing decoherence. In Fig. S7, we compare our theoretical predictions with the Rabi lineshapes observed in experiment at different N_{loc}, with good agreement by setting the dephasing rate $\gamma_z = 0.35 + 0.009N_{\mathrm{loc}}$. Small deviations are observed at the highest densities approaching 200 atoms per site.

S3.2 Scattering parameters

In Ref. (*28, 37*), the relation between the p-wave interaction and p-wave scattering length was missing a factor of 1/2, with the correct coefficient being $3\pi\hbar^2 b_{\alpha\beta}^3/2M$. Using the past notation, in Ref. (*37*) the p-wave scattering lengths were found to be: $\tilde{b}_{eg}^{+} = (-169\pm 23)a_0$, $\tilde{b}_{ee} = (-119 \pm 18)a_0$. These values can be corrected by solving: $b_{ee}^3 - b_{gg}^3 = 2(\tilde{b}_{ee}^3 - b_{gg}^3)$ and $(b_{eg}^{+})^3 - b_{gg}^3 = 2((\tilde{b}_{eg}^{+})^3 - b_{gg}^3)$, which gives $b_{eg}^{+} = (-215.9 \pm 28.2)a_0$ and $b_{ee} = (-155.8 \pm 21.1)a_0$. For p-wave inelastic scattering length β_{ee}, one can multiply the factor $2^{1/3}$ to the value in Ref. (*37*), which gives $\beta_{ee} = (152.5 \pm 16.4)a_0$. Using these corrected values of the p-wave parameters, combined with the measured s-wave scattering lengths in Ref. (*41*), as well as the universal relation between the complex s-wave scattering length $A = a - i\alpha$ and the complex p-wave scattering volume $B^3 = b^3 - i\beta^3$ for a single van der Waals potential (*37, 43*), one can finally obtain Table 1 that includes the updated s-wave and p-wave scattering lengths that are used in this work.

Table 1: ^{87}Sr s-wave and p-wave scattering lengths in Bohr radius (a_0)

Channel	s-wave	p-wave
gg	96.2 ± 0.1	74.5 ± 0.3
eg^+	161.3 ± 2.5	-215.9 ± 28.2
eg^-	69.1 ± 0.9	-41.3 ± 2.7
ee (elastic)	176.3 ± 9.5	-155.8 ± 21.1
ee (inelastic)	17.3^{+14}_{-8}	152.5 ± 16.4

S3.3 Corrections in the tunneling rate from Gaussian beam geometry

In previous sections, we assume a separable confinement potential and tunneling only along the direction of gravity. However in the experimental system, the Gaussian geometry of the laser beams inevitably couple the axial and radial wave functions. This coupling leads to corrections in the nearest-neighbor tunneling rate which now depends on the thermal distribution of the radial modes. Notice that the Gaussian beam profile of a 1D lattice leads to the following trapping potential,

$$V(X,Y,Z) = V_0 - V_0 \cos^2(k_L Z) \exp[-2(X^2+Y^2)/w_L^2], \tag{S47}$$

where $k_L = 2\pi/\lambda_L$ is the lattice wave number, w_L is the beam waist, and $V_0 > 0$ is the lattice depth. Expanding the trapping potential to second order of X, Y, we have

$$V(X,Y,Z) \approx \left[V_0 - \frac{1}{2}M\omega_R^2(X^2+Y^2)\right]\sin^2(k_L Z) + \frac{1}{2}M\omega_R^2(X^2+Y^2), \tag{S48}$$

where the radial trapping frequency is given by $\omega_R = \sqrt{4V_0/Mw_L^2}$. Based on Eq. (S48), an atomic gas with radial temperature T_r feels an effective lattice depth given by $V_0 - k_B T_r$. Although $k_B T_r \ll V_0$, it may still lead to non-negligible corrections to the nearest-neighbor tunneling rate, which shows exponential dependence on lattice depth. Note that

in the large-spin Hamiltonian discussed in previous sections, the interaction parameters are determined by thermal average over radial modes. To take into account the leading order effects of the thermal distribution, we replace the ground band tunnel coupling by

$$J_0(T_r) \approx \frac{4}{\sqrt{\pi}} E_{rec}^{1/4} (V_0 - k_B T_r)^{3/4} \exp\left[-2\sqrt{\frac{V_0 - k_B T_r}{E_{rec}}} \right]. \tag{S49}$$

This correction leads to $\sim 40\%$ increase of nearest-neighbor s-wave interaction strength near the zero-crossing point.

S4 Data File 1

The data presented in Figs. 1-4 is available for download as a separate supplementary file.

REFERENCES AND NOTES

1. S. L. Campbell, R. B. Hutson, G. E. Marti, A. Goban, N. Darkwah Oppong, R. L. McNally, L. Sonderhouse, J. M. Robinson, W. Zhang, B. J. Bloom, J. Ye, A fermi-degenerate three-dimensional optical lattice clock. *Science* **358**, 90–94 (2017).

2. E. Oelker, R. B. Hutson, C. J. Kennedy, L. Sonderhouse, T. Bothwell, A. Goban, D. Kedar, C. Sanner, J. M. Robinson, G. E. Marti, D. G. Matei, T. Legero, M. Giunta, R. Holzwarth, F. Riehle, U. Sterr, J. Ye, Demonstration of 4.8×10^{-17} stability at 1s for two independent optical clocks. *Nat. Photonics* **13**, 714–719 (2019).

3. T. L. Nicholson, S. L. Campbell, R. B. Hutson, G. E. Marti, B. J. Bloom, R. L. McNally, W. Zhang, M. D. Barrett, M. S. Safronova, G. F. Strouse, W. L. Tew, J. Ye, Systematic evaluation of an atomic clock at 2×10^{-18} total uncertainty. *Nat. Commun.* **6**, 6896 (2015).

4. A. D. Ludlow, M. M. Boyd, J. Ye, E. Peik, P. O. Schmidt, Optical atomic clocks. *Rev. Mod. Phys.* **87**, 637–701 (2015).

5. W. F. McGrew, X. Zhang, R. J. Fasano, S. A. Schäffer, K. Beloy, D. Nicolodi, R. C. Brown, N. Hinkley, G. Milani, M. Schioppo, T. H. Yoon, A. D. Ludlow, Atomic clock performance enabling geodesy below the centimetre level. *Nature* **564**, 87–90 (2018).

6. T. Bothwell, C. J. Kennedy, A. Aeppli, D. Kedar, J. M. Robinson, E. Oelker, A. Staron, J. Ye, Resolving the gravitational redshift across a millimetre-scale atomic sample. *Nature* **602**, 420–424 (2022).

7. X. Zheng, J. Dolde, V. Lochab, B. N. Merriman, H. Li, S. Kolkowitz, Differential clock comparisons with a multiplexed optical lattice clock. *Nature* **602**, 425–430 (2022).

8. P. Lemonde, P. Wolf, Optical lattice clock with atoms confined in a shallow trap. *Phys. Rev. A* **72**, 033409 (2005).

9. I. Dimitrova, N. Jepsen, A. Buyskikh, A. Venegas-Gomez, J. Amato-Grill, A. Daley, W. Ketterle, Enhanced superexchange in a tilted mott insulator. *Phys. Rev. Lett.* **124**, 043204 (2020).

10. R. C. Brown, R. Wyllie, S. B. Koller, E. A. Goldschmidt, M. Foss-Feig, J. V. Porto, Two-dimensional superexchange-mediated magnetization dynamics in an optical lattice. *Science* **348**, 540–544 (2015).

11. S. Trotzky, P. Cheinet, S. Fölling, M. Feld, U. Schnorrberger, A. M. Rey, A. Polkovnikov, E. A. Demler, M. D. Lukin, I. Bloch, Time-resolved observation and control of superexchange interactions with ultracold atoms in optical lattices. *Science* **319**, 295–299 (2008).

12. H. Miyake, G. A. Siviloglou, C. J. Kennedy, W. C. Burton, W. Ketterle, Realizing the harper hamiltonian with laser-assisted tunneling in optical lattices. *Phys. Rev. Lett.* **111**, 185302 (2013).

13. M. Aidelsburger, M. Atala, M. Lohse, J. T. Barreiro, B. Paredes, I. Bloch, Realization of the hofstadter hamiltonian with ultracold atoms in optical lattices. *Phys. Rev. Lett.* **111**, 185301 (2013).

14. M. Aidelsburger, M. Lohse, C. Schweizer, M. Atala, J. T. Barreiro, S. Nascimbène, N. R. Cooper, I. Bloch, N. Goldman, Measuring the chern number of hofstadter bands with ultracold bosonic atoms. *Nat. Phys.* **11**, 162–166 (2015).

15. C. J. Kennedy, W. C. Burton, W. C. Chung, W. Ketterle, Observation of Bose-Einstein condensation in a strong synthetic magnetic field. *Nat. Phys.* **11**, 859–864 (2015).

16. J. Simon, W. S. Bakr, R. Ma, M. E. Tai, P. M. Preiss, M. Greiner, Quantum simulation of antiferromagnetic spin chains in an optical lattice. *Nature* **472**, 307–312 (2011).

17. F. Meinert, M. J. Mark, E. Kirilov, K. Lauber, P. Weinmann, A. J. Daley, H.-C. Nägerl, Quantum quench in an atomic one-dimensional ising chain. *Phys. Rev. Lett.* **111**, 053003 (2013).

18. S. Scherg, T. Kohlert, P. Sala, F. Pollmann, B. Hebbe Madhusudhana, I. Bloch, M. Aidelsburger, Observing non-ergodicity due to kinetic constraints in tilted Fermi-Hubbard chains. *Nat. Commun.* **12**, 4490 (2021).

19. E. Guardado-Sanchez, A. Morningstar, B. M. Spar, P. T. Brown, D. A. Huse, W. S. Bakr, Subdiffusion and heat transport in a tilted two-dimensional Fermi-Hubbard system. *Phys. Rev. X* **10**, 011042 (2020).

20. W. Morong, F. Liu, P. Becker, K. S. Collins, L. Feng, A. Kyprianidis, G. Pagano, T. You, A. V. Gorshkov, C. Monroe, Observation of Stark many-body localization without disorder. *Nature* **599**, 393–398 (2021).

21. Q. Beaufils, G. Tackmann, X. Wang, B. Pelle, S. Pelisson, P. Wolf, F. P. dos Santos, Laser controlled tunneling in a vertical optical lattice. *Phys. Rev. Lett.* **106**, 213002 (2011).

22. G. M. Tino, Testing gravity with cold atom interferometry: Results and prospects. *Quantum Sci. Technol.* **6**, 024014 (2021).

23. V. Xu, M. Jaffe, D. Panda Cristian, L. Kristensen Sofus, W. Clark Logan, H. Müller, Probing gravity by holding atoms for 20 seconds. *Science* **366**, 745–749 (2019).

24. G. K. Campbell, M. M. Boyd, J. W. Thomsen, M. J. Martin, S. Blatt, M. D. Swallows, T. L. Nicholson, T. Fortier, C. W. Oates, S. A. Diddams, N. D. Lemke, P. Naidon, P. Julienne, J. Ye, A. D. Ludlow, Probing interactions between ultracold fermions. *Science* **324**, 360–363 (2009).

25. A. D. Ludlow, N. D. Lemke, J. A. Sherman, C. W. Oates, G. Quéméner, J. von Stecher, A. M. Rey, Cold-collision-shift cancellation and inelastic scattering in a Yb optical lattice clock. *Phys. Rev. A* **84**, 052724 (2011).

26. M. D. Swallows, M. Bishof, Y. Lin, S. Blatt, M. J. Martin, A. M. Rey, J. Ye, Suppression of collisional shifts in a strongly interacting lattice clock. *Science* **331**, 1043–1046 (2011).

27. N. D. Lemke, J. von Stecher, J. A. Sherman, A. M. Rey, C. W. Oates, A. D. Ludlow, *p*-wave cold collisions in an optical lattice clock. *Phys. Rev. Lett.* **107**, 103902 (2011).

28. M. J. Martin, M. Bishof, M. D. Swallows, X. Zhang, C. Benko, J. von-Stecher, A. V. Gorshkov, A. M. Rey, J. Ye, A quantum many-body spin system in an optical lattice clock. *Science* **341**, 632–636 (2013).

29. A. M. Rey, A. V. Gorshkov, C. V. Kraus, M. J. Martin, M. Bishof, M. D. Swallows, X. Zhang, C. Benko, J. Ye, N. D. Lemke, A. D. Ludlow, Probing many-body interactions in an optical lattice clock. *Ann. Phys.* **340**, 311–351 (2014).

30. S. Kolkowitz, S. L. Bromley, T. Bothwell, M. L. Wall, G. E. Marti, A. P. Koller, X. Zhang, A. M. Rey, J. Ye, Spin–orbit-coupled fermions in an optical lattice clock. *Nature* **542**, 66–70 (2017).

31. S. L. Bromley, S. Kolkowitz, T. Bothwell, D. Kedar, A. Safavi-Naini, M. L. Wall, C. Salomon, A. M. Rey, J. Ye, Dynamics of interacting fermions under spin–orbit coupling in an optical lattice clock. *Nat. Phys.* **14**, 399–404 (2018).

32. M. L. Wall, A. P. Koller, S. Li, X. Zhang, N. R. Cooper, J. Ye, A. M. Rey, Synthetic spin-orbit coupling in an optical lattice clock. *Phys. Rev. Lett.* **116**, 035301 (2016).

33. J. Zhang, G. Pagano, P. W. Hess, A. Kyprianidis, P. Becker, H. Kaplan, A. V. Gorshkov, Z. X. Gong, C. Monroe, Observation of a many-body dynamical phase transition with a 53-qubit quantum simulator. *Nature* **551**, 601–604 (2017).

34. K. Xu, Z.-H. Sun, W. Liu, Y.-R. Zhang, H. Li, H. Dong, W. Ren, P. Zhang, F. Nori, D. Zheng, H. Fan, H. Wang, Probing dynamical phase transitions with a superconducting quantum simulator. *Sci. Adv.* **6**, eaba4935 (2020).

35. J. A. Muniz, D. Barberena, R. J. Lewis-Swan, D. J. Young, J. R. K. Cline, A. M. Rey, J. K. Thompson, Exploring dynamical phase transitions with cold atoms in an optical cavity. *Nature* **580**, 602–607 (2020).

36. A. Chu, J. Will, J. Arlt, C. Klempt, A. M. Rey, Simulation of *xxz* spin models using sideband transitions in trapped bosonic gases. *Phys. Rev. Lett.* **125**, 240504 (2020).

37. X. Zhang, M. Bishof, S. L. Bromley, C. V. Kraus, M. S. Safronova, P. Zoller, A. M. Rey, J. Ye, Spectroscopic observation of $\mathrm{SU}(N)$-symmetric interactions in Sr orbital magnetism. *Science* **345**, 1467–1473 (2014).

38. S. Smale, P. He, B. A. Olsen, K. G. Jackson, H. Sharum, S. Trotzky, J. Marino, A. M. Rey, J. H. Thywissen, Observation of a transition between dynamical phases in a quantum degenerate Fermi gas. *Sci. Adv.* **5**, eaax1568 (2019).

39. S. Blatt, J. W. Thomsen, G. K. Campbell, A. D. Ludlow, M. D. Swallows, M. J. Martin, M. M. Boyd, J. Ye, Rabi spectroscopy and excitation inhomogeneity in a one-dimensional optical lattice clock. *Phys. Rev. A* **80**, 052703 (2009).

40. W. M. Itano, J. C. Bergquist, J. J. Bollinger, J. M. Gilligan, D. J. Heinzen, F. L. Moore, M. G. Raizen, D. J. Wineland, Quantum projection noise: Population fluctuations in two-level systems. *Phys. Rev. A* **47**, 3554–3570 (1993).

41. A. Goban, R. B. Hutson, G. E. Marti, S. L. Campbell, M. A. Perlin, P. S. Julienne, J. P. D'Incao, A. M. Rey, J. Ye, Emergence of multi-body interactions in a fermionic lattice clock. *Nature* **563**, 369–373 (2018).

42. A. V. Gorshkov, M. Hermele, V. Gurarie, C. Xu, P. S. Julienne, J. Ye, P. Zoller, E. Demler, M. D. Lukin, A. M. Rey, Two-orbital $SU(N)$ magnetism with ultracold alkaline–Earth atoms. *Nat. Phys.* **6**, 289–295 (2010).

43. Z. Idziaszek, P. S. Julienne, Universal rate constants for reactive collisions of ultracold molecules. *Phys. Rev. Lett.* **104**, 113202 (2010).
